# International recommendations for personalised selective internal radiation therapy of primary and metastatic liver diseases with yttrium-90 resin microspheres

**DOI:** 10.1007/s00259-020-05163-5

**Published:** 2021-01-12

**Authors:** Hugo Levillain, Oreste Bagni, Christophe M. Deroose, Arnaud Dieudonné, Silvano Gnesin, Oliver S. Grosser, S. Cheenu Kappadath, Andrew Kennedy, Nima Kokabi, David M. Liu, David C. Madoff, Armeen Mahvash, Antonio Martinez de la Cuesta, David C. E. Ng, Philipp M. Paprottka, Cinzia Pettinato, Macarena Rodríguez-Fraile, Riad Salem, Bruno Sangro, Lidia Strigari, Daniel Y. Sze, Berlinda J. de Wit van der veen, Patrick Flamen

**Affiliations:** 1 Department of Nuclear Medicine, Jules Bordet Institute, Université Libre de Bruxelles, Rue Héger-Bordet 1, B-1000 Brussels, Belgium; 2 Nuclear Medicine Unit, Santa Maria Goretti Hospital, Latina, Italy; 3 Nuclear Medicine, University Hospitals Leuven and Nuclear Medicine & Molecular Imaging, Department of Imaging and Pathology, KU Leuven, Leuven, Belgium; 4 Department of Nuclear Medicine, Hôpital Beaujon, AP-HP.Nord, DMU DREAM and Inserm U1149, Clichy, France; 5 Institute of Radiation Physics, Lausanne University Hospital and University of Lausanne, Lausanne, Switzerland; 6 Department of Radiology and Nuclear Medicine, University Hospital Magdeburg, Germany and Research Campus STIMULATE, Otto-von-Guericke University, Magdeburg, Germany; 7 Department of Imaging Physics, University of Texas MD Anderson Cancer Center, Houston, TX USA; 8 Sarah Cannon Research Institute, Nashville, TN USA; 9 Division of Interventional Radiology and Image Guided Medicine, Department of Radiology and Imaging Sciences, Emory University School of Medicine, Atlanta, GA USA; 10 Department of Radiology, Vancouver General Hospital, University of British Columbia, Vancouver, BC Canada; 11 Department of Radiology and Biomedical Imaging, Yale School of Medicine, New Haven, CT USA; 12 Department of Interventional Radiology, University of Texas MD Anderson Cancer Center, Houston, TX USA; 13 Clinica Universidad de Navarra-IDISNA and CIBEREHD, Pamplona, Spain; 14 Department of Nuclear Medicine and Molecular Imaging, Singapore General Hospital, Singapore, Singapore; 15 Department of Interventional Radiology, Technical University Munich, Munich, Germany; 16 Fondazione IRCCS Ca’ Granda Ospedale Maggiore Policlinico, Milan, Italy; 17 Department of Radiology, Northwestern University, Chicago, IL USA; 18 Department of Medical Physics, IRCCS Azienda Ospedaliero-Universitaria di Bologna, Bologna, Italy; 19 Department of Interventional Radiology, Stanford University School of Medicine, Palo Alto, CA USA; 20 Department of Nuclear Medicine, The Netherlands Cancer Institute, Amsterdam, The Netherlands

**Keywords:** SIRT, Dosimetry, Recommendations, Liver tumours

## Abstract

**Purpose:**

A multidisciplinary expert panel convened to formulate state-of-the-art recommendations for optimisation of selective internal radiation therapy (SIRT) with yttrium-90 (^90^Y)-resin microspheres.

**Methods:**

A steering committee of 23 international experts representing all participating specialties formulated recommendations for SIRT with ^90^Y-resin microspheres activity prescription and post-treatment dosimetry, based on literature searches and the responses to a 61-question survey that was completed by 43 leading experts (including the steering committee members). The survey was validated by the steering committee and completed anonymously. In a face-to-face meeting, the results of the survey were presented and discussed. Recommendations were derived and level of agreement defined (strong agreement ≥ 80%, moderate agreement 50%–79%, no agreement ≤ 49%).

**Results:**

Forty-seven recommendations were established, including guidance such as a multidisciplinary team should define treatment strategy and therapeutic intent (strong agreement); 3D imaging with CT and an angiography with cone-beam-CT, if available, and ^99m^Tc-MAA SPECT/CT are recommended for extrahepatic/intrahepatic deposition assessment, treatment field definition and calculation of the ^90^Y-resin microspheres activity needed (moderate/strong agreement). A personalised approach, using dosimetry (partition model and/or voxel-based) is recommended for activity prescription, when either whole liver or selective, non-ablative or ablative SIRT is planned (strong agreement). A mean absorbed dose to non-tumoural liver of 40 Gy or less is considered safe (strong agreement). A minimum mean target-absorbed dose to tumour of 100–120 Gy is recommended for hepatocellular carcinoma, liver metastatic colorectal cancer and cholangiocarcinoma (moderate/strong agreement). Post-SIRT imaging for treatment verification with ^90^Y-PET/CT is recommended (strong agreement). Post-SIRT dosimetry is also recommended (strong agreement).

**Conclusion:**

Practitioners are encouraged to work towards adoption of these recommendations.

**Supplementary Information:**

The online version contains supplementary material available at 10.1007/s00259-020-05163-5.

## Introduction

Selective internal radiation therapy (SIRT) with yttrium-90 (^90^Y)-loaded microspheres has been broadly adopted as a locoregional therapy for advanced hepatocellular carcinoma (HCC) [1–3], intrahepatic cholangiocarcinoma (ICC) [4, 5], and liver metastases of malignancies including neuroendocrine tumours (NETs) and colorectal cancer (mCRC) [6].

Although SIRT is a well-established therapy, efforts to personalise and refine the planning and administration of therapy are ongoing. The ability to accurately predict, plan and deliver optimal doses to the tumour and non-tumoural tissues, including a final validation of the dose distribution, is a first principle of radiotherapy. Knowing the true absorbed dose to tissue compartments is the primary way to safely individualise therapy for maximal response while respecting normal tissue tolerances. Recent progress in positron emission tomography (PET) imaging has improved the ability to estimate absorbed ^90^Y doses [7–11] and a more accurate dosimetric approach to activity calculation in SIRT is now possible.

Published randomised trials of SIRT were initiated before the widespread introduction of personalised dosimetry approaches, and therefore, expert guidance on how best to perform personalised dosimetry is needed. Recommendations on dosimetry for ^90^Y-glass microspheres for HCC have been published [12], but because of differences in the size and specific activity of ^90^Y-glass microspheres and ^90^Y-resin microspheres, separate recommendations are needed for ^90^Y-resin microspheres. In addition, recommendations should be developed for other tumour types.

Our aim was to provide recommendations to assist practitioners in optimising individualised activity prescription for SIRT with ^90^Y-resin microspheres in primary and metastatic liver tumours. It is anticipated that this manuscript will be the first in a series on this topic that will provide essential guidance for practitioners and future research.

## Methods

**Fig. 1:**
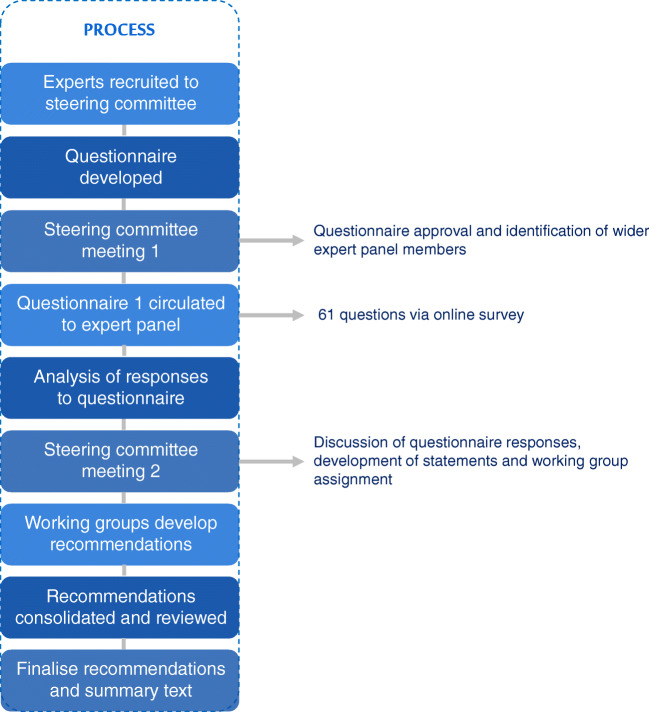
Overview of methodology

The method used to reach agreement was based upon Delphi methods (Fig. 1). The steering committee (SC) consisted of 23 experts in nuclear medicine (*n* = 7), medical physics (*n* = 7), interventional radiology (*n* = 7), radiation/surgical/medical oncology (*n* = 1) and hepatology (*n* = 1) from Europe, North America and Asia. Experts were included based on their recognised clinical expertise, experience with SIRT and academic contributions to the field. Generally, and based on information provided by Sirtex Medical, experts were selected from centres that had conducted over 100 SIRT procedures with ^90^Y-resin microspheres, and if they had published on SIRT and personally been involved in the management of more than 50 patients receiving ^90^Y-resin microspheres.

A questionnaire to collect opinion on pre-SIRT simulation, interventional strategy, individual activity prescription methods and treatment evaluation, was tested and refined by the SC. The finalised questionnaire consisting of 61 questions (Supplementary File 1) was administered anonymously to a broader expert panel of 41 members (including the SC; of the additional 18 included in the expert panel, specialties were nuclear medicine (*n* = 11), medical physics (*n* = 2), interventional radiology (*n* = 4) and radiation/surgical/medical oncology (*n* = 1)). Upload of the questionnaire, collection and collation of the responses was managed by a third-party agency. Questionnaire responses were refined into a series of statements and the level of agreement of responders was rated (‘strong agreement’ when ≥ 80% of responders agreed with a statement; ‘moderate agreement’ when 50–79% of responders agreed with a statement; these definitions were developed by the SC based on the range of definitions of consensus used in Delphi studies [13]). Responses were not assessed/compared by responder specialty. Working groups from the SC summarised the evidence to support sub-groups of these statements. Published data on SIRT dosimetry from blinded or prospective randomised controlled trials are limited, and most evidence cited in this recommendation would be considered weak. Using an evidence grading system such as the GRADE system [14] is therefore unlikely to add value to these recommendations.

## Results

A summary of all responses is provided in the Supplementary File 2. Centres at which the expert panel members practiced had a median of 14 years experience with ^90^Y-resin microspheres and more than 50% of centres conducted more than 40 SIRT procedures each year. The recommendations derived from the questionnaire responses are summarised below into those related to interventional strategy and pre-treatment considerations (Table 1), individual activity prescriptions (Table 2) and treatment evaluation (Table 3).

**Table 1:** Recommendations on the interventional strategy and pre-treatment ^99m^Tc-MAA simulation when planning SIRT with ^90^Y-resin microspheres

Recommendation number	Recommendation	Strength of agreement
General pre-treatment considerations and the multidisciplinary tumour board		
R1	Treatment strategy and therapeutic intent should be defined by a multidisciplinary team	Strong
R2	When available, whole body FDG PET/CT (for FDG-avid tumours) or Octreotate-PET/CT (for neuroendocrine tumours) should be performed in addition to the SIRT work-up procedure to assess presence of extrahepatic disease	Strong
R3	The arterial liver anatomy should be assessed before simulation	Strong
R4	Underlying liver function should be determined by clinical scoring (Child-Pugh, ALBI, etc.)	Strong
R5	In cases of bi-lobar manifestation of the tumour, a single injection into the common hepatic artery is not recommended. A same day bi-lobar procedure (left and right hepatic artery separately in a single session) may be recommended depending on individual characteristics, such as liver function, treatment intent and practical considerations, such as the ease of patient visit	Moderate
R6	When staged (separate days) bi-lobar infusion is used, a period of 3–8 weeks should be left between the two treatments	Strong
CT angiography		
R7	When available, cone-beam CT is useful for the identification of vessel targeting for SIRT	Strong
R8	Cone-beam CT may also be useful for checking tumour perfusion, volumetric analysis for activity prescription and extrahepatic deposition assessment	Moderate
R9	Conventional cross-sectional imaging (CT or MRI) are options for volumetric analysis before SIRT	Moderate
^99m^Tc-MAA scintigraphic imaging		
R10	Scintigraphic imaging of ^99m^Tc-MAA is recommended before SIRT for identification of intra- and extrahepatic depositions, assessment of lung shunt, for calculation of the activity to be injected and volumetric analysis of the treatment field	Strong
R11	^99m^Tc-MAA or cone-beam CT are both useful for extrahepatic deposition verification	Strong
R12	SPECT/CT is the recommended imaging method for evaluating ^99m^Tc-MAA distribution within the liver	Strong
R13	Tumours should be delineated on conventional cross-sectional images and correlated with ^99m^Tc-MAA images	Moderate
R14	Conventional cross-sectional imaging (CT or MRI) and ^99m^Tc-MAA SPECT/CT are all options for volumetric analysis before SIRT	Moderate
R15	The portion of a tumour with complete absence of vascularisation on perfusion CT/CBCT and/or metabolic activity on [^18^F]FDG PET/CT could be excluded from the target volume (and the healthy liver volume), consideration of the portion depends upon activity prescription calculation method	Moderate
R16	Generally, SIRT should be withheld for lesions with less ^99m^Tc-MAA uptake than non-tumoural liver. In exceptional situations, SIRT may be appropriate, for example, when ablative SIRT is possible and in other clinical scenarios (i.e. if it is still possible to selectively deliver a significant amount of radiation to the lesion)	Moderate
R17	SIRT should be conducted as soon as possible after the simulation and no more than 4 weeks after simulation	Strong
R18	If a staged (separate days) bi-lobar approach is planned, the need for a repeat of the simulation is greater with a greater interval between the two SIRT sessions. However, no clear agreement was reached on whether staged simulation should be recommended or not, and if staged simulation is performed, there was no agreement on whether or not to recommend performing the second simulation during the same session as the first SIRT	None
R19	There is no consensus on whether the ^99m^Tc-MAA simulation should be re-performed if the catheter position is modified or when additional embolisation is needed.	None
Lung shunt estimation		
R20	Planar imaging should be used, as a minimum, for evaluating the lung shunt with ^99m^Tc-MAA. SPECT/CT may be used to supplement this in selected cases	Moderate

*ALBI*, albumin-bilirubin; *CT*, computed tomography; *FDG*, fluorodeoxyglucose; *MRI*, magnetic resonance imaging; *PET*, positron emission tomography; *SIRT*, selective internal radiation therapy; *SPECT*, single-photon emission computed tomography; ^*99m*^*Tc-MAA*, technetium-99 m labelled macroaggregated albumin

**Table 2:** Individual activity prescription recommendations for the use of SIRT with ^90^Y-resin microspheres

Recommendation number	Recommendation	Strength of agreement
Activity prescription methods		
R21	A personalised approach to activity prescription is recommended when whole liver SIRT is planned and when selective non-ablative treatment is planned. The partition model (MIRD-based) or 3D dosimetry (voxel-based) are recommended, but the safety of these methods is still unproven	Strong
R22	Likewise, when doing selective ablative treatment, an activity prescription method is needed and a personalised approach to activity prescription is recommended	Strong
Personalised activity prescription methods (MIRD-based/voxel-based)		
R23	For selective ablative treatments, it is recommended to consider a higher specific activity, hence a lower number of microspheres. A high T/N ratio warrants the consideration of a higher specific activity	Strong
R24	In the absence of a better surrogate, it is recommended to determine the T/N ratio from signal distribution evaluated from ^99m^Tc-MAA SPECT/CT	Strong
Lung shunt management		
R25	It is recommended that lung shunt limits are expressed as the calculated absorbed radiation dose (Gy) resulting from the administered activity (this does not exclude the use of percentages to express lung shunt limits)	Strong
R26	On planar scans, recommended cut-off values for lung exposure are 30 Gy (single) and 50 Gy (cumulative)	Moderate
R27	This is preferred to expressing cut-offs as a percentage, if percentages are used, a cut-off of 20% is recommended	None
R28	Measuring the patient-specific lung mass for assessing dose to lung tissue is recommended when LSF is close to the recommended cut-offs (when LSF is not close to cut-offs, the assumption of 1 kg lung mass is acceptable)	Moderate
Safety dose cut-off—whole liver/bi-lobar treatment		
R29	When patients have a ‘non-compromised’ liver, the recommended mean absorbed dose limit for safety to non-tumoural liver is 40 Gy, when doing whole liver treatment. When the liver is heavily pretreated or when there is suspicion of compromised liver function, this cut-off should be reduced to 30 Gy but should be estimated on an individualised basis	Strong
Safety dose cut-off—lobar and segmental treatment		
R30	There was no clear agreement on whether to use the same absorbed dose safety limits for unilobar treatment as used for whole liver treatment, most experts would not	None
R31	For unilobar or segmental treatment, when the volume and function of the contralateral liver lobe is sufficient (FLR cut-off of the contralateral liver lobe of 30–40%), a more aggressive treatment (than for whole liver treatment) may be useful (depending on several factors such as the intent of treatment, liver function and tumour type)	Strong
R32	In unilobar or segmental treatment, if the function of the treated lobe is to be preserved, a mean absorbed dose cut-off of 40 Gy is proposed. In cases where some loss of function is acceptable, a higher cut-off could be used	Moderate
R33	There was no clear agreement on whether to perform a more aggressive unilobar treatment in cirrhotic patients	None
Safety dose cut-off—lobectomy and segmentectomy		
R34	In lobectomy a mean absorbed dose to the non-tumoural liver of > 70 Gy for ablative therapy is proposed	Strong
R35	A higher mean absorbed dose should be used for segmentectomy—possibly > 150 Gy	Strong
Safety dose cut-off—SIRT before surgery		
R36	The minimal time window between SIRT and surgery should be defined by monitoring liver volumetry/function and tumour control, while considering the decay of ^90^Y which will reach safe levels after 1 month	Moderate
Efficacy dose cut-off		
R37	To target tumour ablation/complete response, a minimum mean absorbed dose cut-off of 100–120 Gy is proposed for mCRC	Strong
R38	To yield a response, a minimum mean absorbed dose cut-off of 100–120 Gy is proposed for HCC	Strong
R39	To yield a response, a minimum mean absorbed dose cut-off of 100–120 Gy is proposed for ICC	Moderate
R40	To yield a response, a minimum mean absorbed dose cut-off of 100–150 Gy is proposed for NET	Moderate

*CT*, computed tomography; *FLR*, future liver remnant; *HCC*, hepatocellular carcinoma; *ICC*, intrahepatic cholangiocellular carcinoma; *LSF*, lung shunt fraction; *mCRC*, metastatic colorectal cancer; *MIRD*, Medical Internal Radiation Dose; *NET*, neuroendocrine tumour; *SIRT*, selective internal radiation therapy; *SPECT*, single-photon emission computed tomography; ^*99m*^*Tc-MAA*, technetium-99 m labelled macroaggregated albumin; *T/N*, tumour/normal liver

**Table 3:** Treatment evaluation recommendations for the use of SIRT with ^90^Y-resin microspheres

Recommendation number	Recommendation	Strength of agreement
Treatment verification		
R41	It is important to verify that the position/location of the catheter is the same during SIRT as it was during the ^99m^Tc-MAA simulation by visually comparing the positions on angiography	Strong
R42	Post-SIRT residual activity of microspheres in the vial, tubing system and syringe should be measured	Strong
R43	Post-SIRT imaging for treatment verification is used for dosimetry and visual verification	Strong
R44	Post-SIRT imaging for treatment verification is used for future (re)-SIRT	Moderate
R45	Post-SIRT imaging should be performed using the best option available—it should be visual and quantitative and therefore ^90^Y-PET is preferred (when ^90^Y-PET is not available, BECT is an acceptable alternative—but is difficult to use to get quantitative verification)	Strong
R46	Post-SIRT dosimetry is recommended	Strong
Treatment response evaluation		
R47	When post-SIRT imaging and/or dosimetry shows areas of possible insufficient treatment of the tumour, it is recommended to wait for follow-up response imaging before deciding on the need to re-treat	Moderate

*BECT*, ^90^Y bremsstrahlung emission computed tomography; *PET*, positron emission tomography; *SIRT*, selective internal radiation therapy; ^*99m*^*Tc-MAA*, technetium-99 m labelled macroaggregated albumin

### Interventional strategy and pre-treatment ^99m^Tc-MAA simulation

#### General pre-treatment considerations and the multidisciplinary tumour board

Personalised SIRT needs a holistic view of the patient and the disease. The disease stage, long-term and immediate treatment aims, and morphological and biological characteristics of the tumour and the surrounding liver, should all be considered when establishing a SIRT treatment plan. As such, the continuous exchange of information and opinions between multiple specialties is required (R1, Table 1). The multidisciplinary tumour board (MDT) should, as a minimum, consist of the clinician overseeing the care of the patient (medical oncologist, radiation oncologist, hepatologist, surgeon, others), the team that will perform the treatment (e.g. interventional radiologist, nuclear medicine specialist, medical physicist, radiation oncologist and surgeon) and any other specialty that may provide useful information (e.g. diagnostic radiologist or pathologist).

SIRT may be useful for liver-only disease and may also be recommended in selected cases when extrahepatic disease is present and not deemed prognostically relevant. Therefore, whole body imaging to detect extrahepatic disease is important to exclude patients from SIRT or guide their management plan [15] (R2, Table 1).

Additional essential pre-SIRT steps for all tumour types (whether or not the liver is (pre)cirrhotic) include assessment of the arterial liver anatomy, underlying liver function and portal hypertension (R3, R4, Table 1).

When there is bi-lobar manifestation of the tumour, a same day bi-lobar approach to SIRT may be useful to provide more flexibility than single-injection whole liver SIRT (R5, Table 1). There is no rationale for a staged (separate days) bi-lobar approach. However, if this approach is chosen based upon individual factors such as treatment intent, a period of 3–8 weeks should be left between the two treatments (R6, Table 1).

#### Cone-beam CT angiography

There is evidence that cone-beam computed tomography (CBCT) may be useful for vessel targeting and may identify feeding branches to tumours that CT or magnetic resonance imaging (MRI) fail to detect [16] (R7, Table 1). Therefore, if available, CBCT is recommended to complement CT or MRI. Additionally, CBCT is useful for providing reliable information on extrahepatic arterial perfusion, and is helpful for differentiating areas of segmental perfusion and confirming full tumour coverage from the site of infusion [16, 17] (R7, R8, Table 1). However, CT and MRI remain valuable options for volumetric analysis before SIRT, and CT can be considered a minimum standard [18] (R9, Table 1). Hybrid CT/angiography is preferred to CBCT where available.

#### ^99m^Tc-MAA scintigraphic imaging

Given the similar median size of macroaggregated albumin (MAA) particles and resin microspheres [19], MAA distribution pattern serves as a surrogate for how ^90^Y-resin microspheres will localise (R10, Table 1). While ^99m^Tc-MAA acts as a reasonably accurate surrogate, it does have limitations and discrepancies between pre- and post-SIRT dose estimates can occur due to several factors including flow differences between MAA and resin microspheres, catheter position deviations and differences between imaging modalities used [20, 21]. During pre-treatment angiography, a calibrated amount of ^99m^Tc-MAA is administered at selected sites within the hepatic arterial tree. As MAA degrades rapidly in the liver [22] scintigraphy should start ≤ 1 h after administration.

Abdominal extrahepatic depositions identified on scintigraphy are caused by physiological accumulation of dissociated ^99m^Tc-pertechnetate (which can hinder accurate evaluation of the gastric region) or ^99m^Tc-MAA lodging in tissues. To limit dissociation, ^99m^Tc-MAA should be prepared under strict quality control and sodium perchlorate may be given to reduce gastric pertechnetate uptake. Focal gastrointestinal or pancreatic uptake is important as it may lead to severe radiation damage during ^90^Y-SIRT. Other sites include the gall bladder, the abdominal wall (through the falciform artery) and the hilar hepatic artery. Single-photon emission computed tomography/CT (SPECT/CT) has been shown to be more effective than planar imaging for identifying extrahepatic uptake sites [23]. It is recommended to identify and, if possible, remedy the vascular source of extrahepatic uptake, and to use angiographic imaging such as CBCT, before proceeding with treatment (R7, R8, R11, Table 1).

Intrahepatic ^99m^Tc-MAA distribution should be evaluated using SPECT/CT, instead of planar scintigraphy or SPECT alone (R12, Table 1), and ideally shows focal uptake at all tumour sites within the treatment field, with limited uptake in the non-tumoural liver parenchyma. Scatter and attenuation correction will improve both visual and quantitative SPECT evaluation. Compensation of attenuation can be done on planar images using geometric mean of antero-posterior views The degree of uptake in non-tumoural parenchyma is less relevant in the case of ablative segmentectomy, other low volume targets or in hypertrophy-inducing lobectomy. Conventional cross-sectional/metabolic images are used to identify tumour volume and should be correlated with ^99m^Tc-MAA images to improve delineations and report on areas of the tumour with limited or no uptake (R13, R14, Table 1). The portion of a tumour with complete absence of vascularisation on perfusion CT/CBCT and/or metabolic activity on [^18^F]fluorodeoxyglucose (FDG) PET/CT could be excluded from the target volume (R15, Table 1). ^99m^Tc-MAA SPECT/CT is used to quantify uptake in tumour lesions and normal parenchyma for the purpose of activity calculation [24, 25]. Therefore, SIRT should generally be withheld for lesions with ^99m^Tc-MAA uptake that is similar to, or less than, non-tumoural liver, and when there is a lack of enhancement on CBCT (R16, Table 1). In a limited number of cases, especially in mCRC, there is low concentration of ^99m^Tc-MAA despite rim hypervascularisation on CBCT. These cases should not be excluded from treatment even if the ^99m^Tc-MAA cannot be used for predictive dosimetry.

To limit anatomical/vascularisation modification caused by disease progression, treatment should be conducted as soon as possible after the simulation (R17, Table 1). When staged bi-lobar SIRT is used, performing staged (before each SIRT) simulation is not mandatory, the initially obtained lung shunt fraction can be carried over to the second treatment (R18, Table 1). The position/location of the catheter during the administration of ^90^Y-microspheres should be consistent with the position during the ^99m^Tc-MAA simulation [26]. When a segmental treatment is planned, it is essential that the catheter position is in the same arterial branch. When segmental treatment is planned, lobar ^99m^Tc-MAA simulation may be performed, for example, to avoid damage to the segmental artery. Clinical justification for adjustment or alteration of catheter position between sessions should be documented. The need to re-perform ^99m^Tc-MAA simulation when the catheter position is modified may depend on the degree of position change; slight differences in catheter tip position, especially near vascular bifurcations, can induce major differences in hepatic distribution (R19, Table 1).

#### Lung shunt fraction estimation

The estimated lung shunt fraction (eLSF) represents the fraction of injected microspheres lodged within the pre-capillary bed of the lungs and can be estimated on images from the ^99m^Tc-MAA simulation, either planar or SPECT/CT (R20, Table 1), by dividing the counts of the lung by the sum of the counts in the lung and liver. This estimation is biased by two types of error: (1) some of the MAA particles are smaller than the resin microspheres and intrahepatic degradation of ^99m^Tc-MAA leads to lower liver and higher lung counts, increasing eLSF [22]; and (2) physical factors, such as volume averaging of the liver dome into the lung compartment during respiration, lower attenuation in lung versus liver tissue and scatter of liver activity into the lung, also increase eLSF. SPECT/CT with attenuation and scatter correction can reduce the latter error [27].

### Individual activity prescription

#### Activity prescription methods

Personalised therapeutic activity prescription in SIRT aims to maximise tumour response while sparing non-target tissues from undesired toxicity by tailoring the treatment according to patient-specific parameters (e.g. local activity and dose deposition, and tissue masses and functionality) (R21, R22, Table 2). The need for treatment personalisation is supported by several publications [4, 28–33], and is in compliance with the principle of optimisation expressed in the COUNCIL DIRECTIVE 2013/59/EURATOM Article 56 [34].

##### (Modified) body surface area method

If personalised therapeutic activity prescription is feasible, it is preferred to the body surface area (BSA) method. Several studies demonstrated the lack of personalisation of the BSA method leading to under/overtreatment, and therefore, to poorer outcome when compared to more personalised approaches such as the partition model [4, 28, 35]. The safe use of modified BSA (mBSA), when a more selective treatment (e.g. lobar) is performed, has nevertheless been confirmed in a number of prospective trials.

##### Personalised activity prescription methods (MIRD-based/voxel-based) (R23, R24, Table 2)

Personalised activity prescription relies on dosimetry that considers the patient-specific anatomy and perfusion of microspheres [36]. According to the Medical Internal Radiation Dose (MIRD) formalism, the absorbed dose under equilibrium to a compartment *D*_*C*_, knowing the administered ^90^Y-activity *A*_*C*_ in that compartment and its mass *M*_*C*_ is calculated by:

1 $$ {D}_c\left[ Gy\right]=\frac{49.67\times {A}_c\left[\mathrm{GBq}\right]}{M_c\left[\mathrm{kg}\right]} $$

The partition model considers the distribution of microspheres into the lungs, the tumour and the non-tumoural liver, by 3D quantification on SPECT or SPECT/CT images [37–39]. The first estimation of the activity *A* to administer would then be calculated from the targeted dose to tumour *D*_*T*_ by [40]:

2 $$ A\left[\mathrm{GBq}\right]=\frac{D_T\left[\mathrm{Gy}\right]\times \left({M}_N\left[\mathrm{kg}\right]+{M}_T\left[\mathrm{kg}\right]\times r\right)}{49.67\times r\times \left(1-L\right)} $$

with L being the lung shunt fraction:

3 $$ L=\frac{\mathrm{total}\ \mathrm{counts}\ \mathrm{in}\ \mathrm{lungs}\ }{\ \mathrm{total}\ \mathrm{counts}\ \mathrm{in}\ \mathrm{lungs}+\kern0.5em \mathrm{total}\ \mathrm{counts}\ \mathrm{in}\ \mathrm{liver}} $$

and *r* the tumour to normal liver ratio (T/N ratio):

4 $$ r=\frac{\mathrm{average}\ \mathrm{counts}\ \mathrm{per}\ \mathrm{ml}\ \mathrm{in}\ \mathrm{tumour}}{\mathrm{average}\ \mathrm{counts}\ \mathrm{per}\ \mathrm{ml}\ \mathrm{in}\ \mathrm{non}-\mathrm{tumoural}\ \mathrm{liver}} $$

where *M*_*N*_ and *M*_*T*_ being the masses of non-tumoural liver and tumour, respectively.

MIRD equations and partition model can be adapted to consider as many compartments as needed (i.e. for bi-lobar or segmental approaches) and by using 3D quantification. Using the activity obtained with Eq. 2, the absorbed dose to the considered compartments should be computed (Eq. 1), and if needed, the activity to administer should be adapted to respect the different safety/efficacy dose limits (with the primary consideration being the safety limits). A further degree of personalisation is voxel-based dosimetry, where each voxel is considered as a source and/or a target, allowing visualisation of 3D absorbed dose distributions [29, 41, 42] and the evaluation of degree of heterogeneity in both organs and targets through the dose volume histograms (DVHs) [43].

#### Individual activity prescription

##### Lung shunt management

During the ^99m^Tc-MAA simulation, the eLSF allows (1) calculation of the absorbed dose to the lung parenchyma and (2) compensating prescribed treatment activity for shunted activity to prevent underdosing in target regions. The main safety purpose is prevention of radiopneumonitis, which can be fatal. The use of eLSF to define maximum radiation doses to the lungs has strongly reduced the incidence of radiopneumonitis; 2 cases out of 1022 treated patients in modern large randomised controlled trials that have used planar imaging [1–3, 44, 45]. Thresholds should be expressed as the calculated dose to the lungs (R25, R26, R27 and R28; Table 2). These historical thresholds suffer from methodological issues (described earlier) but are demonstrated to be safe.

##### Safety dose cut-off

1. Whole liver/bi-lobar treatment

A mean absorbed dose to non-tumoural liver of ≤ 40 Gy is considered safe (R29, Table 2). This dose level can be derived from external beam radiotherapy (EBRT), using biological effective doses [43]. Based on ^90^Y-bremsstrahlung emission CT (BECT) images, a liver dose of 52 Gy (95% CI 44–61 Gy) in a whole liver injection provided a 50% probability of ≥ G2 liver toxicity in patients with HCC [31]. Tolerability of SIRT depends on the initial liver function (Child-Pugh score or baseline bilirubin) [46]. Therefore, when the liver is heavily pretreated or when there is suspicion of compromised liver function, the cut-off should be reduced (R29, Table 2).

2) Lobar and segmental treatment

In unilobar or segmental treatments, a more aggressive treatment (i.e. higher mean absorbed dose to non-tumoural parenchyma) can be considered when some loss of function due to treatment is acceptable (but not when function is to be preserved), but a cut-off was not agreed (R30, R31, R32, Table 2). In these treatment approaches, voxel-based modelling of the absorbed dose based on ^99m^Tc-MAA distribution may help to predict the radiation-induced effects throughout the liver. Currently, DVHs allow estimation of the tissue volume fraction receiving a minimum dose threshold.

3) Lobectomy and segmentectomy

Radiation lobectomy, with the intent to induce contralateral lobe hypertrophy while achieving tumour control and including a biologic test of time, may be considered in patients with unilobar disease and a small anticipated future liver remnant, in an attempt to facilitate curative surgical resection. While there is some evidence for a mean absorbed dose cut-off to achieve this, further validation is needed (R34, Table 2) [47–49].

Radiation segmentectomy may be considered for localised disease (≤ 2 segments) supplied by a segmental artery, and unamenable for other curative therapies because of the tumour localisation or patient comorbidities. The small volume of the liver treated allows administration of high mean absorbed doses to produce tumour ablation with low toxicity risk to the untreated parenchyma, but evidence is limited (R35, Table 2).

4) SIRT before surgery

SIRT before surgery is well tolerated [50, 51]. The time window between SIRT and surgery depends on tumour biology, ^90^Y decay and treatment aim (R36, Table 2).

##### Efficacy dose cut-off

Heterogeneity exists among reported dose-outcome relationships because of the variability of the applied outcome measure (Table 4), which include (1) benefit to the patient increased (progression-free survival/overall survival (OS)/quality of life); (2) local tumour response to the treatment anatomic response (RECIST) and metabolic response on [^18^F]FDG PET (partial or complete reduction of [^18^F]FDG uptake/metabolic volume/total lesion glycolysis [TLG]). Therefore, efficacy dose cut-offs should always be considered in the context of the applied outcome measurement. OS is currently the de facto clinical endpoint. Importantly, when metabolic response is the endpoint, cut-offs maximising probabilities of complete metabolic response should be prioritised for treatment planning.

**Table 4:** Key studies on dose-response with ^90^Y-resin microspheres

Study	Population	Activity prescription method	Lesion dosimetry assessment	Response assessment	Results
van den Hoven et al. 2016 [28]	Chemorefractory mCRC (*n* = 30)	BSA	^90^Y-PET 3D voxel-based	Tumour-absorbed dose quantified on ^90^Y-PET versus TLG on ^18^F-FDG PET	50% reduction in TLG at 1 month associated with prolonged OS At least 40–60 Gy required to achieve 50% reduction in TLG
Levillain et al. 2018 [29]	Liver-only mCRC progressing after chemotherapy (*n* = 24)	Partition model	^90^Y-PET 3D voxel-based	TLG for each target lesion measured on FDG PET/CT	Cut-offs of 39 Gy and 60 Gy predict non-metabolic response and high-metabolic response, respectively
Willowson et al. 2017 [30]	Unresectable mCRC progressing despite chemotherapy (*n* = 22)	Modified BSA	^90^Y-PET 3D voxel-based	Peak standardised uptake value and TLG	Approximately 50 Gy derived as the critical threshold for a significant response (> 50% reduction in TLG)
Stigari et al. 2010 [31]	Unresectable HCC (*n* = 73)	BSA	^90^Y-BECT 3D voxel-based	CR and PR according to RECIST	Median dose to achieve CR/PR was 99 Gy
Hermann et al. 2020 [32]	Locally advanced unresectable HCC (*n* = 121)	BSA	^99m^Tc-MAA SPECT 3D voxel-based	Retrospective assessment of OS in group receiving tumour radiation-absorbed dose < 100 Gy or ≥ 100 Gy	Median OS 14.1 month in those receiving ≥ 100 Gy Median OS 6.1 months in those receiving < 100 Gy
Garin et al. 2019 [52]	HCC with PVT	Multiple	MIRD and 3D voxel-based	Review of studies using treatment response and OS	Predictor of response and OS with a threshold of 100–120 Gy
Levillain et al. 2019 [4]	Unresectable and chemorefractory ICC (*n* = 58)	BSA or partition model	^99m^Tc-MAA SPECT 3D voxel-based	OS	Median OS was 5.5 months when BSA used (mean radiation dose to tumour of 38 Gy) Median OS was 14.9 months when partition model was used (mean radiation dose to tumour of 86 Gy)
Chansanti et al. 2017 [33]	Unresectable mNET (*n* = 15)	Partition model	^99m^Tc-MAA SPECT MIRD	CR and PR according to mRECIST	Cut-off of ≥ 191.3 Gy for tumour-specific absorbed dose predicted tumour response with 93% specificity < 72.8 Gy predicted non-response with 100% specificity

*BSA*, body surface area; *CR*, complete response; *CT*, computed tomography; *FDG*, fluorodeoxyglucose; ^*99m*^*Tc-MAA*, technetium-99 m labelled macroaggregated albumin; *HCC*, hepatocellular carcinoma; *ICC*, intrahepatic cholangiocarcinoma; *mCRC*, metastatic colorectal cancer; *mNET*, metastatic neuroendocrine tumour; *OS*, overall survival; *PET*, positron emission tomography; *BECT*, ^90^Y bremsstrahlung emission computed tomography; *PR*, partial response; *TLG*, total lesion glycolysis

Another source of variability in the dose-outcome assessment stems from the different activity prescription methods used in different studies with many using BSA/mBSA models. Similarly, the reported tumour-absorbed doses may be based on ^99m^Tc-MAA images collected in the treatment planning or may be based upon post-SIRT ^90^Y-PET/CT.

1. Liver dominant colorectal cancer metastases

Several prospective and retrospective studies reported the existence of a lesion-based dose-response relationship (Table 4). Post-SIRT tumour-absorbed dose cut-offs of 60 Gy for predicting a metabolic response (defined as > 50% reduction of TLG) were reported (using partition model) [29], and doses > 50 Gy (using mBSA method) [30] and > 40–60 Gy (using BSA method) [28] provided better responses in two studies using a similar endpoint. In these studies, lesions that received more than 100–120 Gy had a higher probability of complete metabolic response (R37, Table 2).

2) Hepatocellular carcinoma

Several studies on pre- and post-therapy imaging indicate the recommended threshold tumour dose [31, 52, 53] (R38, Table 2). In the SARAH trial (using BSA method), a post hoc analysis of putative delivered dose based on ^99m^Tc-MAA SPECT/CT showed that OS and disease control were significantly better with a tumour-absorbed dose ≥ 100 Gy [32]. The probability of disease control at 6 months was 72% (95% CI 46–89%) and 81% (95% CI 58–93%) with a tumour-absorbed dose of 100 Gy and 120 Gy, respectively. The probability for tumour control increased when there was good concordance between pre-therapy ^99m^Tc-MAA SPECT/CT and post-therapy BECT or PET/CT.

3) Cholangiocarcinoma

There are few publications dealing with SIRT efficacy [54, 55], and only one [4] with tumour-absorbed dose, in patients with unresectable ICC. In particular, there are no reports of the absorbed dose threshold associated with tumour control. However, Levillain et al. showed that median OS (14.9 vs 5.5 months) and mean tumour-absorbed doses (86 vs 38 Gy) were significantly higher when therapeutic activity prescription was based on partition model compared to BSA method [4]. In the absence of robust evidence, our recommendation is based on the experience and data obtained from centres participating in the questionnaire (R39, Table 2).

4) Neuroendocrine tumours

Patients with NETs have particular features that distinguish them from other patients eligible for SIRT: (1) absence of underlying liver disease, (2) relatively long OS and (3) pronounced hypervascular tumours with high T/N ratios on ^99m^Tc-MAA and post-therapy imaging [56]. There is a paucity of data regarding dose-response relationships in NET. Using partition model, a preliminary dose-response relationship was reported between ^99m^Tc-MAA SPECT/CT and mRECIST-based response in 55 lesions in 15 patients—a higher mean tumour dose resulted in a better response rate (207 vs 114 Gy, in responders vs non-responders, respectively) [33]. Response rate was 96% when the tumour dose was > 191 Gy. No response was seen with a tumour dose < 73 Gy. Our recommendation is based on providing a sufficient dose to tumour while limiting the dose to healthy parenchyma to avoid long-term complications (R40, Table 2).

### Treatment evaluation

#### Treatment verification

With catheter-directed therapies, it is important to verify that the position/location of the catheter during the ^99m^Tc-MAA simulation is consistent with the position during the administration of ^90^Y-microspheres [26] (R41, Table 3). However, factors such as flow, perfusion and nonlaminar hydrodynamics limit the ability to optimally reproduce position and flow dynamics. As a minimum, fluoroscopic reproduction of the catheter position should be performed during all administrations.

Post-SIRT residual activity of microspheres should be measured to determine the actual administered activity (R42, Table 3). There was moderate agreement on how to achieve this; the most popular method was to determine the mean dose rate of the delivery system before and after treatment. Other options include quantitative imaging by ^90^Y-PET/CT or BECT, or measuring residual activity within each injection material using a dose calibrator.

Post-SIRT imaging for qualitative and quantitative assessment is highly recommended to address two fundamental aspects (R43, R44, Table 3). Firstly, it allows the verification of the treatment to the intended territory. Identifying technical failure with lack of uptake in the target liver parenchyma and/or in selected lesions allows consideration of additional therapies in a timely manner [57]. Secondly, it serves to detect any possible extrahepatic activity, which can cause serious complications, such as ulceration and gastrointestinal bleeding [58]. Knowledge of microsphere deposition in non-target areas may guide appropriate actions to minimise possible radio-induced toxicity. Post-SIRT imaging of ^90^Y distribution may be performed using ^90^Y-PET/CT or BECT [59, 60]. Many studies have shown qualitatively superior resolution and contrast with ^90^Y-PET/CT compared to BECT, and ^90^Y-PET/CT can be easily used for quantification, supporting the use of ^90^Y-PET/CT as the preferred post-SIRT imaging technique [8, 10, 61] (R45, Table 3). However, when ^90^Y-PET is not available, BECT is an acceptable alternative to visually assess dose distribution [31].

Post-SIRT image-based dosimetry is recommended (R46, Table 3) to verify and evaluate agreement between planned and delivered dose. Post-SIRT dosimetry can help to assess the robustness of planned dose constraints, and to identify novel and more robust dose constraints guaranteeing the efficacy and safety of treatment [43, 62]. To correlate doses with patient outcomes, quantitative imaging with ^99m^Tc-MAA SPECT/CT and/or ^90^Y-PET/CT is mandatory. As with all nuclear/radiology imaging, local acquisition, reconstruction and data analysis must be validated to provide quantitative accuracy and system recovery.

#### Treatment response evaluation

Clinical and biochemical assessment after SIRT for any significant side effects is typically performed at 1–2 months post-SIRT. Imaging assessment of tumour response should be at 1–3 months post-SIRT, and every 2–3 months thereafter. The clinically relevant ‘treatment response’, and thus the most suitable imaging technique, is defined differently depending on the type of tumour (e.g. variable FDG avidity) and treatment goal. In a pre-operative setting when bridging to surgery, complete metabolic response and/or tumour shrinkage (depending on tumour type) is the goal and high definition anatomo-metabolic imaging techniques are recommended (PET/CT/MRI), often needing longer follow-up. In a non-curative setting, functional imaging techniques (PET/MRI) indicating treatment resistance and early progression are recommended in order to rapidly identify the need for potential additional therapy. If there is possible insufficient treatment of the tumour, the need to re-treat should be assessed on follow-up (R47, Table 3), and the decision to re-treat earlier should consider the clinical status of the patient, the safety/suitability for re-treatment, and the overall clinical intent of treatment.

### Future directions

Published data on personalised SIRT from blinded or prospective randomised controlled trials are limited. Recently, a randomised trial showed that personalised activity prescription based on ^99m^Tc-MAA SPECT/CT with glass microspheres significantly improved median OS in patients with HCC [63]. Therefore, personalised SIRT with ^90^Y-resin microspheres must be included in future prospective randomised controlled trial designs. In the meantime, these recommendations provide guidance for personalising SIRT with ^90^Y-resin microspheres in primary and metastatic liver cancers, but we acknowledge that the absence of prospective data limit the strength of these recommendations. Furthermore, efforts are needed to provide CE- and/or FDA-approved treatment planning software, dedicated personnel and dosimetry reimbursement, so that personalised SIRT becomes part of clinical routine.

Several developments in SIRT are ongoing, and therefore, were not endorsed in these recommendations. Visual and quantitative assessment of the hepatic function using hepatobiliary scintigraphy and/or MRI-primovist, and post-treatment quantitative dosimetry using BECT images are promising, but more data are needed. As stated in the ‘Introduction’ section, this living document will continue to be updated as new data emerge.

## Conclusion

Personalised activity prescription, based on dosimetry and multidisciplinary management for optimisation of safety and efficacy, is recommended when conducting SIRT with ^90^Y-resin microspheres. Practitioners are encouraged to use these recommendations to perform personalised SIRT with ^90^Y-resin microspheres. This publication is not endorsed by any government entity or professional organisation. Decisions to modify or disregard these recommendations are the responsibility of managing clinicians.

## Supplementary information

Supplementary file 1 Questionnaire associated with the manuscript (DOCX 27 kb)

Supplementary file 2 SURVEY OF BEST PRACTICE -SIRT DOSIMETRY (PDF 449 kb)

Supplementary file 3 video abstract (MP4 454,983 kb)

## Data Availability

Data from the survey used in the current study are available from the corresponding author on reasonable request.
