# Supplementary material for: International recommendations for personalised selective internal radiation therapy of primary and metastatic liver diseases with yttrium-90 resin microspheres
Source: Eur J Nucl Med Mol Imaging. 2021 Jan 12;48(5):1570–84. doi: 10.1007/s00259-020-05163-5 (PMC8113219; doi:10.1007/s00259-020-05163-5)
Supplement: Supplementary file 1 — Questionnaire associated with the manuscript (DOCX 27 kb) [file 259_2020_5163_MOESM1_ESM.docx]

Supplementary file 1 – Questionnaire associated with the manuscript, **International recommendations for personalised selective internal radiation therapy of primary and metastatic liver diseases with yttrium-90 resin microspheres**

| **SURVEY OF BEST PRACTICE - SIRT DOSIMETRY** | | | | |
| --- | --- | --- | --- | --- |
|  | SIRT | SIRT in general using Y-90 |  |  |
|  | MA | Multiple answers possible |  |  |
|  | SA | Single answer only |  |  |
| **N°** | **Type** | **Question** | **Answer** |  |
| 1a | Text | Tell us about your treatment centre a) | Name: |  |
| 1b | Text | Tell us about your treatment centre b) | Country: |  |
| 2 | Text | When did you/your centre start using **resin microspheres for SIRT?** | Year: |  |
| 3a | SA | How many SIRT procedures did you/your institution perform in the year 2017 | 0-15 |  |
|  |  |  | 16-40 |  |
|  |  |  | >40 |  |
| 3b | SA | How many SIRT procedures did you/your institution perform in the year 2018 | 0-15 |  |
|  |  |  | 16-40 |  |
|  |  |  | >40 |  |
| 3c | SA | How many SIRT procedures did you/your institution perform in the year 2019 | 0-15 |  |
|  |  |  | 16-40 |  |
|  |  |  | >40 |  |
| **We ask for your opinion on best practice, even if it is not your daily practice.** | | | | |
| **Pre-SIRT work-up and interventional strategy** | | | | |
| 4 | MA | Should SIRT be limited to liver-only disease? | Yes |  |
|  |  |  | No |  |
|  |  |  | When extrahepatic disease is controlled by additional therapies |  |
|  |  |  | Other (please specify) |  |
| 5 | SA | Should whole body FDG-PET/CT (for FDG-avid tumours) or Octreotate PET-PET/CT (for neuroendocrine tumours) be performed to assess presence of extrahepatic disease? | Yes |  |
|  |  |  | No |  |
|  |  |  | Other (please specify) |  |
| 6 | MA | How should **underlying liver function** be determined? | Clinical scoring (Child-Pugh, ALBI, etc) |  |
|  |  |  | Biopsy of non-tumoural liver |  |
|  |  |  | Elastography |  |
|  |  |  | Hepatobiliary scintigraphy |  |
|  |  |  | MRI-primovist |  |
|  |  |  | Other (please specify) |  |
| 7 | SA | If available, would you routinely do a BrIDA hepatobiliary (HIDA) scan? | Yes |  |
|  |  |  | No |  |
| 8 | SA | Should the treatment strategy (e.g whole liver/bi-lobar/uni-lobar/selective/super-selective) and therapeutic intent (non-ablative selective/ablative selective/etc) be defined at the multi-disciplinary tumour board? | Yes |  |
|  |  |  | No |  |
|  |  |  | Patient should be re-presented to tumour board after the work-up |  |
|  |  |  | Other (please specify) |  |
| 9 | SA | In case of **bi-lobar** manifestation of tumour, which SIRT strategy do you recommend? | Whole liver infusion in a single session via proper hepatic artery |  |
|  |  |  | Left and right hepatic artery separately in a single session |  |
|  |  |  | Sequential left and then right(or vice versa) |  |
|  |  |  | Other (please specify) |  |
| 10 | SA | When **sequential bi-lobar infusion** is recommended, how long do you wait between the two treatments? | 0-1 week |  |
|  |  |  | 1-2 weeks |  |
|  |  |  | 3-8 weeks |  |
|  |  |  | Other (please specify) |  |
| 11 | SA | When **sequential bi-lobar infusion**, is recommended should the treatment simulation be performed sequentially as well? | Yes |  |
|  |  |  | No |  |
|  |  |  | Other (please specify) |  |
| 11a | SA | ***If yes***: do you recommend performing the second simulation during the **same session** as the first treatment? I.e., in total 3 angiography procedures: 1st : simulation 2nd: 1st treatment and 2nd simulation 3rd: 2nd treatment | Yes |  |
|  |  |  | No |  |
|  |  |  | Other (please specify) |  |
| 12 | SA | Do you recommend assessing the arterial liver anatomy before simulation? | Yes |  |
|  |  |  | No |  |
| 13 | MA | What is the rationale for the evaluation using ^99m^Tc-MAA before SIRT? | Lung shunt assessment |  |
|  |  |  | Extrahepatic deposition assessment (e.g., intestines) |  |
|  |  |  | Intrahepatic assessment (tumour targeting) |  |
|  |  |  | Volumetric analysis |  |
|  |  |  | Calculation of activity needed |  |
|  |  |  | Other (please specify or comment) |  |
| 14 | MA | Which imaging method should be used to evaluate the **lung-shunt** with ^99m^Tc-MAA? | Planar |  |
|  |  |  | SPECT |  |
|  |  |  | SPECT–CT |  |
|  |  |  | Planar and SPECT(-CT) |  |
| 15 | MA | Which imaging method should be used to evaluate ^99m^Tc-MAA **distribution within the liver**? | Planar |  |
|  |  |  | SPECT |  |
|  |  |  | SPECT–CT |  |
|  |  |  | Other (please specify) |  |
| 16 | SA | Should tumour(s) be directly delineated on ^99m^Tc-MAA images? | Yes |  |
|  |  |  | No, tumour(s) must be delineated on diagnostic images |  |
|  |  |  | Other (please specify or comment) |  |
| 17 | MA | When there is less ^99m^Tc-MAA uptake in the lesion than the healthy liver, should SIRT be withheld for that lesion? | Yes |  |
|  |  |  | No, when ablative SIRT is possible |  |
|  |  |  | Other (please specify or comment) |  |
| 18 | MA | When should cone-beam CT be used for SIRT? | For extrahepatic deposition assessment |  |
|  |  |  | To check tumour perfusion |  |
|  |  |  | For volumetric analysis for activity prescription |  |
|  |  |  | Identification of vessel targeting |  |
| 19 | MA | Which imaging method do you recommend for **extra-hepatic deposition verification?** | ^99m^Tc-MAA SPECT |  |
|  |  |  | ^99m^Tc-MAA SPECT-CT |  |
|  |  |  | Cone-beam CT |  |
|  |  |  | ^99m^Tc-MAA planar |  |
| 20 | MA | Which imaging method do you recommend for **volumetric analysis**? | ^99m^Tc-MAA SPECT |  |
|  |  |  | ^99m^Tc-MAA SPECT-CT |  |
|  |  |  | Cone-beam CT |  |
|  |  |  | ^99m^Tc-MAA Planar |  |
|  |  |  | MRI |  |
|  |  |  | CT (diagnostic) |  |
| 21 | SA | What is the maximum time one should allow between simulation and SIRT? | 0-2 weeks |  |
|  |  |  | 3-4 weeks |  |
|  |  |  | >4weeks |  |
|  |  |  | Not important |  |
| 22 | SA | Should the necrotic portion of a tumour be included in the target volume? | Yes |  |
|  |  |  | No |  |
|  |  |  | Other (please specify or comment) |  |
| 23 | SA | The simulation has been carried out, the activity to administer has been determined and the treatment is scheduled. For some reason the catheter position has to be changed. Would you recommend re-performing the simulation? | Yes, I would re-perform the simulation |  |
|  |  |  | No |  |
|  |  |  | Only if the perfusion field has changed |  |
|  |  |  | Other (please specify or comment) |  |
| **We ask for your opinion on best practice, even if it is not your daily practice.** | | | | |
| **Individual activity prescription** **methods** | | | | |
| 24 | MA | The multi-disciplinary tumour board decided to perform **whole liver treatment**: which activity prescription method do you recommend? | BSA |  |
|  |  |  | Partition-model (MIRD-based) |  |
|  |  |  | 3D dosimetry (voxel-based) |  |
|  |  |  | Other (please specify or comment) |  |
| 25 | MA | The multi-disciplinary tumour board decided to perform **selective non-ablative treatment**: which activity prescription method do you recommend? | BSA |  |
|  |  |  | Partition-model (MIRD-based) |  |
|  |  |  | 3D dosimetry (voxel-based) |  |
|  |  |  | Other (please specify or comment) |  |
| 26 | SA | The multi-disciplinary tumour board decided to perform **selective ablative treatment**: is an activity prescription method recommended? | Not necessary, just inject until reaching stasis |  |
|  |  |  | Needed in order to administer the correct amount of activity |  |
|  |  |  | Other (please specify or comment |  |
| 26a | MA | ***If needed***: which activity prescription method do you recommend? | BSA |  |
|  |  |  | Partition-model (MIRD-based) |  |
|  |  |  | 3D dosimetry (voxel-based) |  |
|  |  |  | Other (please specify or comment) |  |
| 27 | MA | In which circumstances would you consider higher specific activity of resin microspheres (i.e. specific activity=activity per sphere; classical sphere specific activity is 50 Bq/sphere)? | Whole liver treatment |  |
|  |  |  | Selective non-ablative |  |
|  |  |  | Selective ablative treatment (with or without induction of contralateral hypertrophy) |  |
|  |  |  | Low T/N |  |
|  |  |  | High T/N |  |
|  |  |  | Impaired vasculature |  |
|  |  |  | Other (please specify or comment) |  |
| 28 | SA | Should ^99m^Tc-MAA images be used to determine the tumour to non-tumoural liver uptake ratio ? | Yes |  |
|  |  |  | No |  |
|  |  |  | Depends on type of tumour |  |
|  |  |  | Other (please specify or comment) |  |
| 28a | MA | ***If no***: what imaging technique should be used to for determineing the tumour to non-tumoural liver uptake ratio? | Gadolinium-enhanced MRI angiography |  |
|  |  |  | CT angiography |  |
|  |  |  | Cone-beam CT |  |
|  |  |  | Other (please specify or comment) |  |
| 28b | MA | ***If yes***: how do you recommend estimating the tumour to non-tumoural liver uptake ratio? | Using ROIs on planar images |  |
|  |  |  | Using volume segmentation on SPECT/CT |  |
|  |  |  | Using volume segmentation on multi-dimensional images |  |
|  |  |  | Other (please specify or comment) |  |
| **We ask for your opinion on best practice, even if it is not your daily practice.** | | | | |
| **Individual activity prescription** | | | | |
| **Lung-shunt** | | | | |
| 29 | MA | How should **lung-shunt limits** be expressed? | Shunting activity to lung |  |
|  |  |  | Shunting percentage |  |
|  |  |  | Calculated absorbed radiation dose |  |
|  |  |  | Other (please specify or comment) |  |
| 30 | Text | What cut-off for lung exposure should be used? **PLANAR** | ….. % |  |
|  |  |  | … Gy single |  |
|  |  |  | … Gy cumulative |  |
|  |  |  | Other (please specify or comment) |  |
| 31 | Text | What cut-off for lung exposure should be used for percent or dose-based limit? **SPECT or SPECT/CT** | ….. % |  |
|  |  |  | … Gy single |  |
|  |  |  | … Gy cumulative |  |
|  |  |  | Other (please specify or comment) |  |
| 32 | SA | Would you recommend measuring the lung volume for assessing dose to lung tissue? | Yes |  |
|  |  |  | No |  |
| **Safety: whole liver treatment dose prescription** | | | | |
| 33 | Text | For **whole liver treatment,** what mean absorbed dose limit to non-tumoural liver do you recommend? | ...Gy in normal healthy liver |  |
|  |  |  | … Gy in presence of reduced liver function (e.g., cirrhosis) |  |
|  |  |  | … Gy in heavily pretreated liver (e.g., multiple lines of systemic therapy, surgery, etc) |  |
| **Safety: lobar treatment dose prescription** | | | | |
| 34 | SA | For **uni-lobar treatment**, would you use the same absorbed dose safety limits as for whole liver (bi-lobar) treatment? | Yes |  |
|  |  |  | No |  |
| 35 | SA | For **uni-lobar treatment**, would you perform a more aggressive treatment when the volume and function of the contralateral liver lobe is sufficient? | Yes |  |
|  |  |  | No |  |
|  |  |  | Other (please specify) |  |
| 35a | Text | ***If yes***: which FLR cut-off of contralateral liver lobe should be used? | ….. ml |  |
|  |  |  | …… percentage |  |
|  |  |  | Other (please specify or comment) |  |
| 35b | Text | ***If yes***: what mean absorbed dose cut-off to **non-tumoural l**iver do you recommend? | …Gy |  |
|  |  |  | Other (please specify) |  |
| 35c | SA | ***If yes***: would you also perform a more aggressive treatment in **cirrhotic** patients? | Yes |  |
|  |  |  | No |  |
| 35d | Text | ***If yes***: what mean absorbed dose cut-off to non-tumoural liver do you recommend in **cirrhotic** patients? | … Gy |  |
|  |  |  | Other (please specify) |  |
| **Safety: lobectomy/segmentectomy dose prescription - may also be called A-TARE (Ablative SIRT/transarterial radioembolisation)** | | | | |
| 36 | Text | For **lobectomy/segmentectomy**, what mean absorbed dose to lobe/segment should be used for ablative therapy? | As high as possible: infusion until stasis |  |
|  |  |  | ….Gy mean absorbed dose |  |
|  |  |  | Other (please specify or comment) |  |
| 37 | Text | For **lobectomy/segmentectomy**, if the intention is to induce a contralateral lobe hypertrophy, what would be the recommended mean absorbed dose cut-off to non-tumoural liver within the treated lobe? | As high as possible: infusion until stasis |  |
|  |  |  | ….Gy mean absorbed dose |  |
|  |  |  | Other (please specify or comment) |  |
| 38 | MA | In such a pre-operative SIRT setting, what should be the minimal time window between SIRT and surgery? | 6-8 weeks |  |
|  |  |  | 8-12 weeks |  |
|  |  |  | >12 weeks |  |
|  |  |  | Time to be defined by monitoring of liver volumetry/function and tumour control |  |
|  |  |  | Other (please specify or comment) |  |
| **Efficacy: tumour dose prescription - recommendations of minimum mean absorbed dose to tumour for:** | | | | |
| 39a | Text | **Hepatocellular carcinoma** | … Gy |  |
|  |  |  | Other/Comment…. |  |
| 39b | Text | **Cholangiocarcinoma** | … Gy |  |
|  |  |  | Other/Comment…. |  |
| 39c | Text | **Metastatic colorectal carcinoma** | … Gy |  |
|  |  |  | Other/Comment…. |  |
| 39d | Text | **Metastatic neuroendocrine tumor** | … Gy |  |
|  |  |  | Other/Comment…. |  |
| **We ask for your opinion on best practice, even if it is not your daily practice.** | | | | |
| **Treatment and treatment evaluation** | | | | |
| 40 | MA | Do you think it is important to verify whether the position/location of the catheter is the same **during SIRT as it was during the 99mTc-MAA simulation?** ***If yes***, how? | No, I do not check whether the position is the same |  |
|  |  |  | Yes, I try to recall the injection position from the ^99m^Tc-MAA injection |  |
|  |  |  | Yes, I try to closely mimic the injection position from the ^99m^Tc-MAA injection by visually comparing the positions on angiography |  |
|  |  |  | Yes, other (please specify or comment) |  |
| 41 | MA | Would you determine the post-SIRT residual activity of microspheres in the vial, tubing system, syringe?  ***If yes***, how? | No, I do not determine the residual activity, I assume that all the activity have been administered |  |
|  |  |  | Yes, by measuring the mean dose rate of the injection box before treatment and after (by placing all the injection material inside the box) |  |
|  |  |  | Yes, by measuring the activity within each injection material using a dose calibrator |  |
|  |  |  | Yes, other (please specify or comment) |  |
| 42 | MA | Post-SIRT imaging for treatment verification is used for: | Visual verification |  |
|  |  |  | Detection of extra-hepatic disease |  |
|  |  |  | Dosimetry |  |
|  |  |  | Future (re)-SIRT |  |
|  |  |  | Other (please specify) |  |
| 43 | SA | Post-SIRT imaging for treatment verification is: | Mandatory |  |
|  |  |  | Recommended |  |
|  |  |  | Optional |  |
|  |  |  | Not necessary |  |
|  |  |  | Other (please specify or comment) |  |
| 43a | MA | ***If recommended***, treatment verification should be performed using: | BECT (Bremsstrahlung) |  |
|  |  |  | Y-90 PET |  |
|  |  |  | Other (please specify or comment) |  |
| 43b | SA | ***If recommended***, treatment verification should be: | Visual only |  |
|  |  |  | Visual and quantitative |  |
|  |  |  | Other (please specify or comment) |  |
| 44 | SA | Post-SIRT dosimetry is: | Mandatory |  |
|  |  |  | Recommended |  |
|  |  |  | Optional |  |
|  |  |  | Not necessary |  |
|  |  |  | Other (please specify or comment) |  |
| 45 | SA | If post-SIRT dosimetry shows **underdosing of the tumour**, is retreatment recommended (to increase the cumulative dose to the tumour)? | Yes |  |
|  |  |  | No, I will wait to see follow-up response images and decide whether it is important to retreat |  |
|  |  |  | Other (please specify or comment) |  |
