# Supplementary material for: International recommendations for personalised selective internal radiation therapy of primary and metastatic liver diseases with yttrium-90 resin microspheres
Source: Eur J Nucl Med Mol Imaging. 2021 Jan 12;48(5):1570–84. doi: 10.1007/s00259-020-05163-5 (PMC8113219; doi:10.1007/s00259-020-05163-5)

SURVEY OF BEST PRACTICE - SIRT DOSIMETRY

SurveyMonkey

Q4: How many SIRT procedures did you/your institution perform in the year 2017?

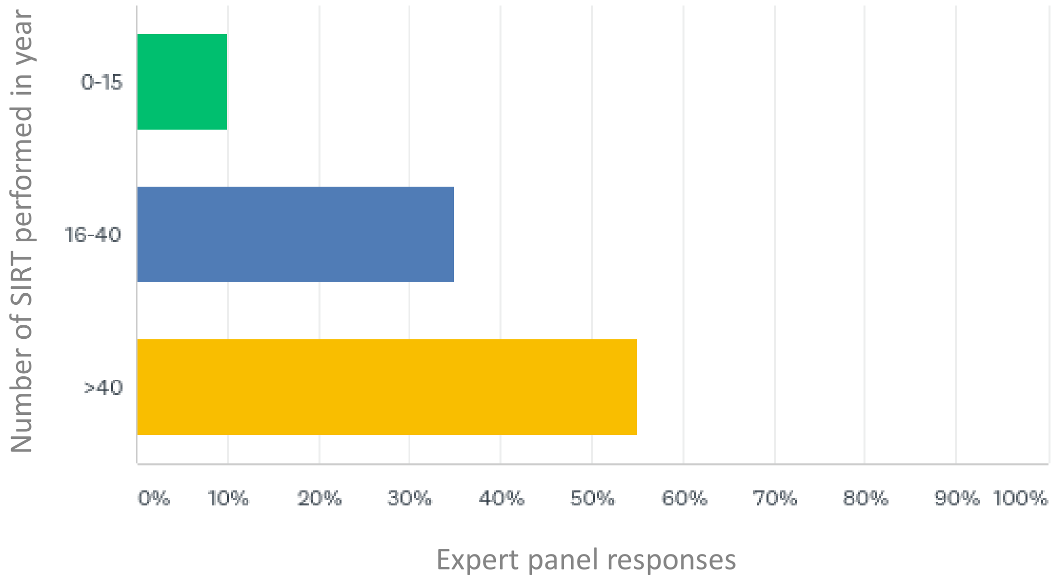

Q5: How many SIRT procedures did you/your institution perform in the year 2018?

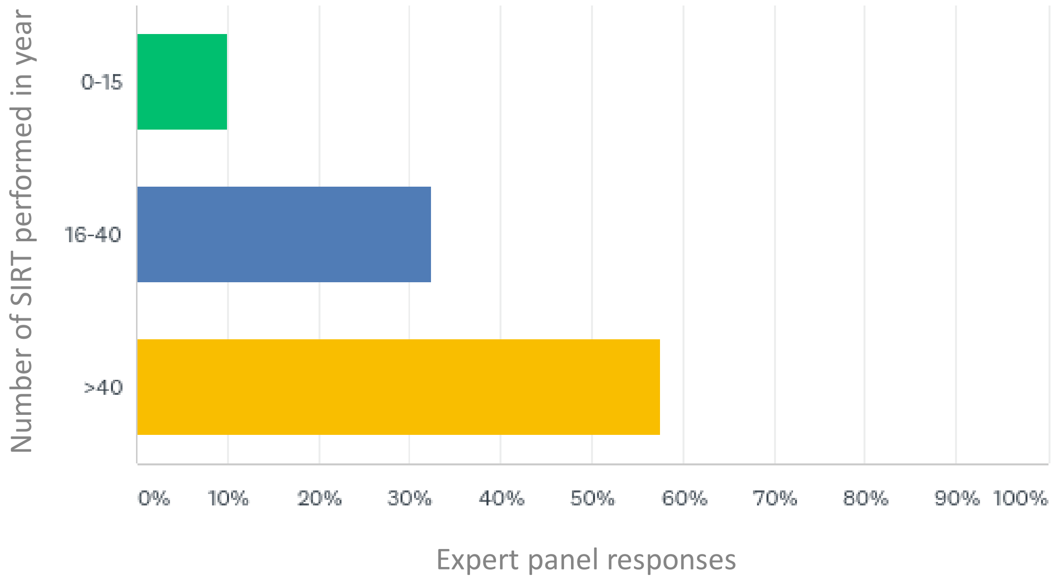

Q6: How many SIRT procedures did you/your institution perform in the year 2019?

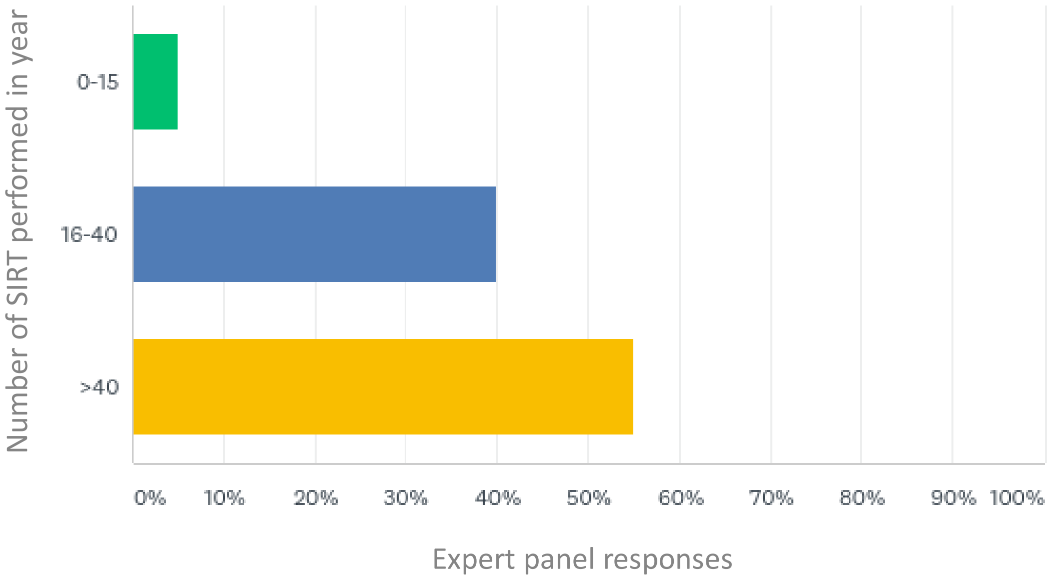

Q7: Should SIRT be limited to liver-only disease?

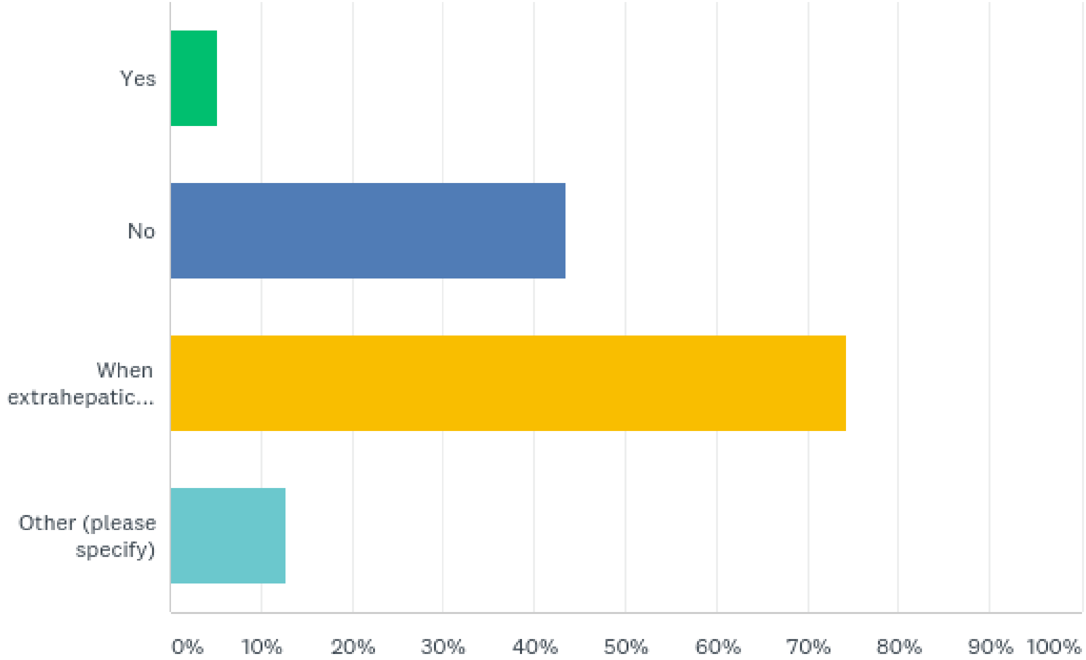

Q8: Should whole body FDG-PET/CT (for FDG-avid tumours) or Octreotate PET-PET/CT (for neuroendocrine tumours) be performed to assess presence of extrahepatic disease?

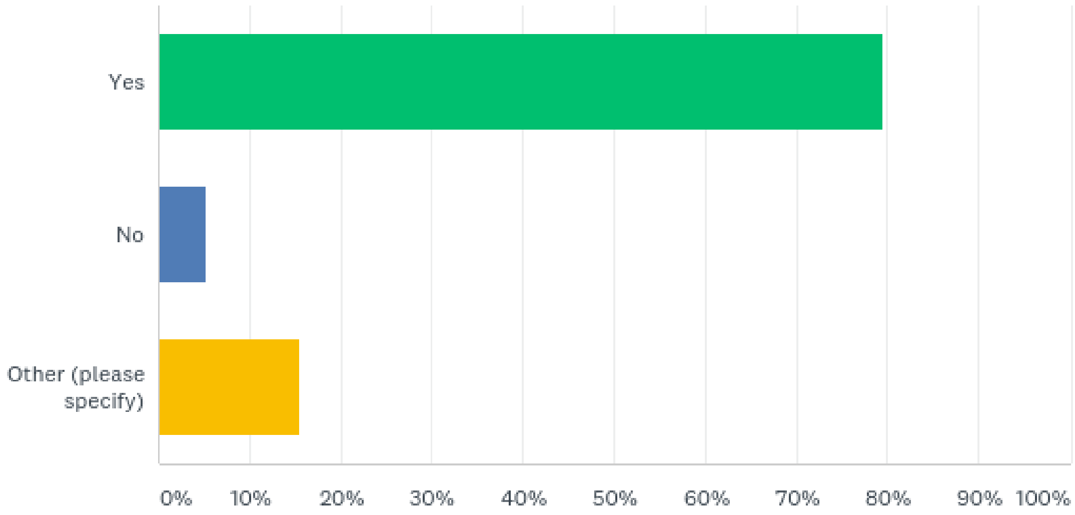

Q9: How should underlying liver function be determined?

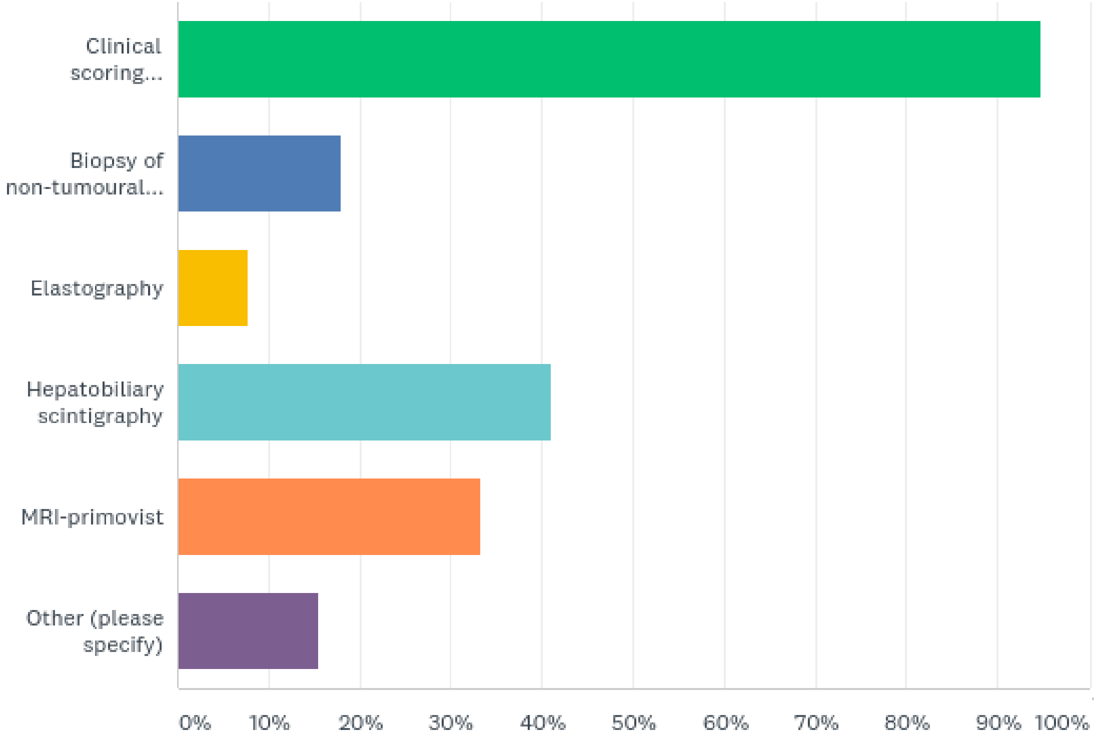

Q10: If available, would you routinely do a BrIDA hepatobiliary (HIDA) scan?

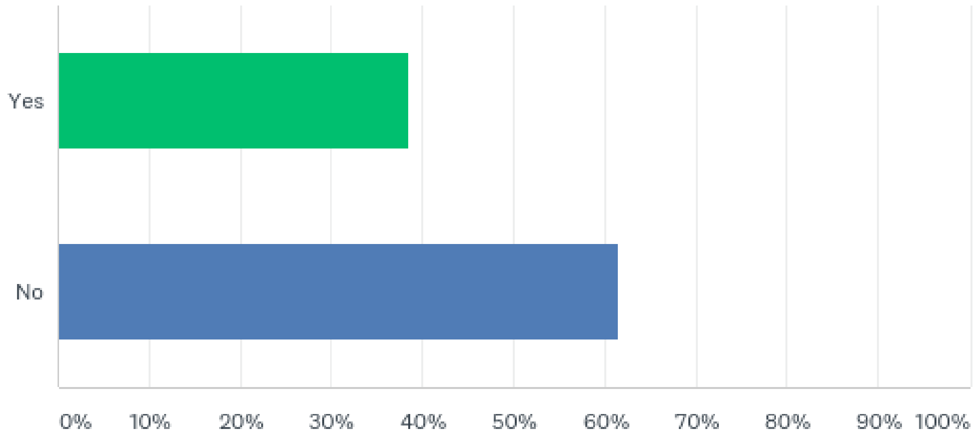

Q11: Should the treatment strategy (e.g whole liver/bi-lobar/uni-lobar/selective/super-selective) and therapeutic intent (non-ablative selective/ablative selective/etc) be defined at the multi-disciplinary tumour board?

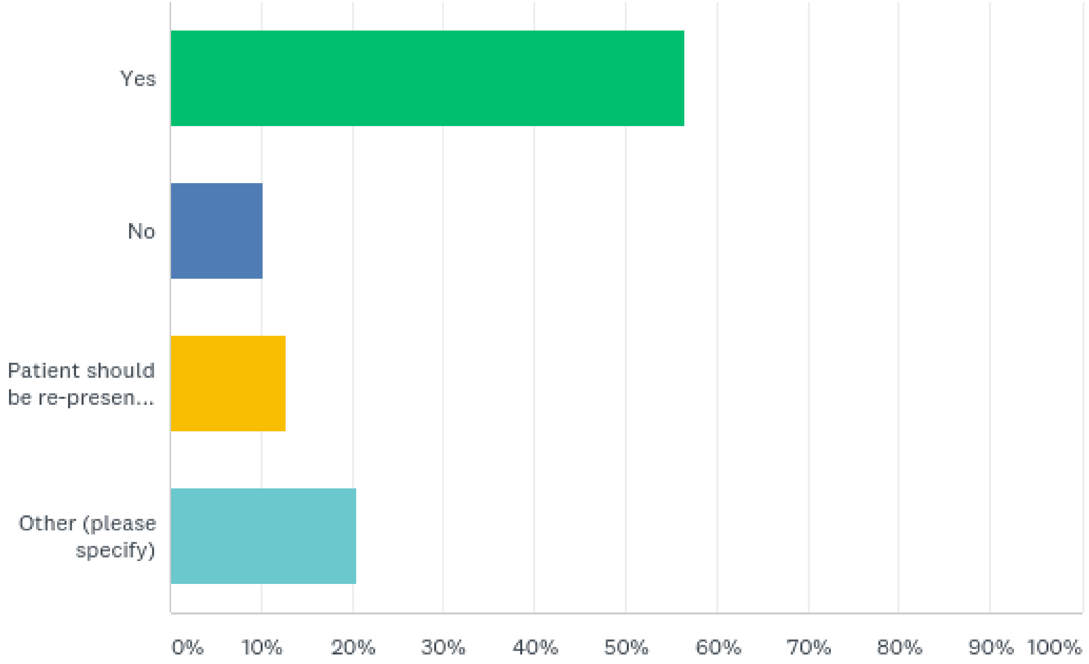

Q12: In case of bi-lobar manifestation of tumour, which SIRT strategy do you recommend?

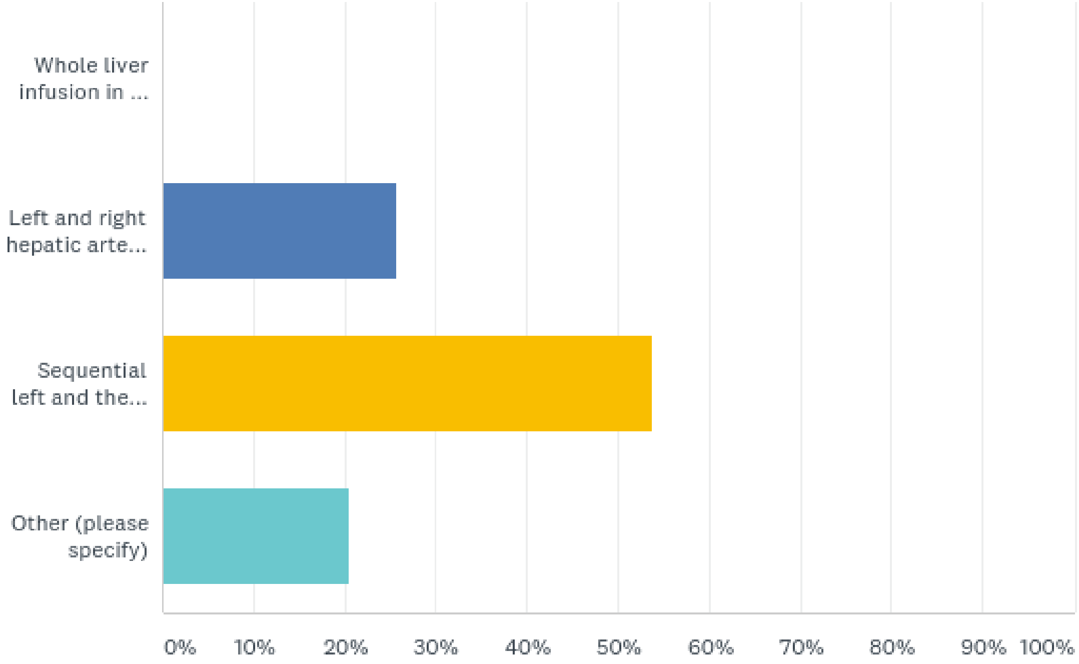

Q13: When sequential bi-lobar infusion is recommended, how long do you wait between the two treatments?

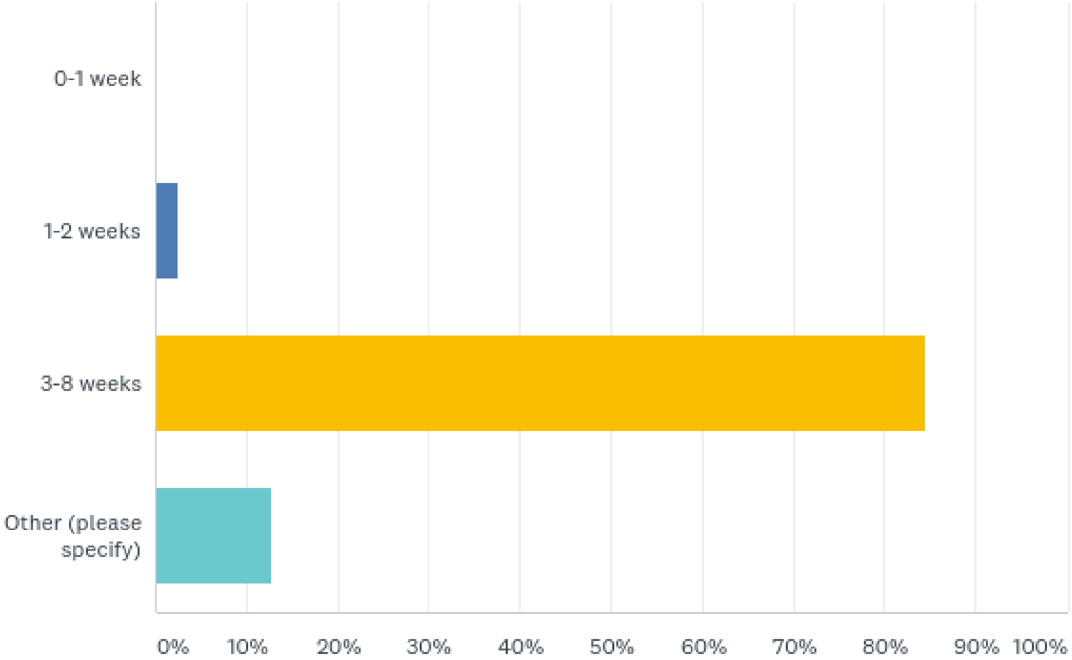

Q14: When sequential bi-lobar infusion is recommended, should the treatment simulation be performed sequentially as well?

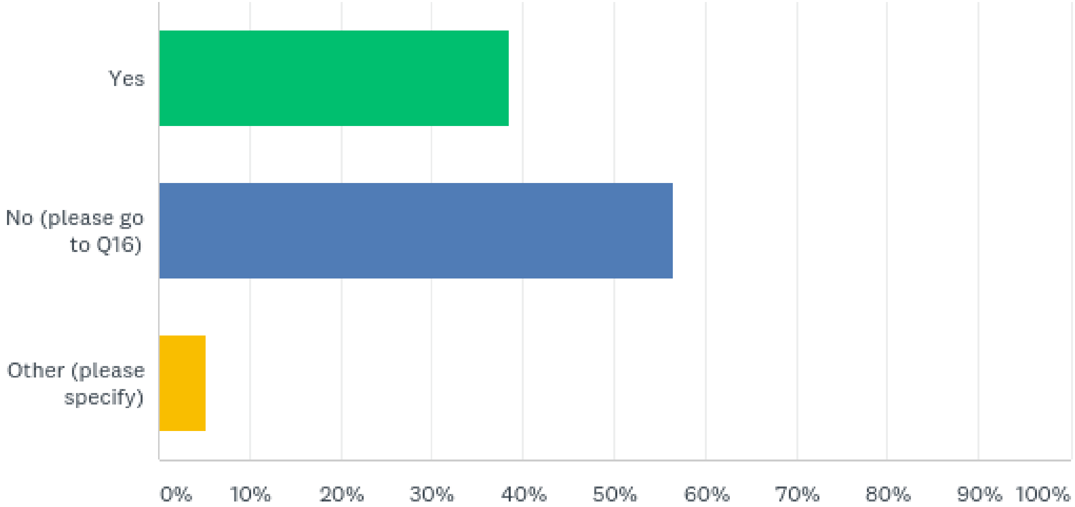

Q15: If you answered 'YES' to Q14: do you recommend performing the second simulation during the same session as the first treatment? I.e., in total 3 angiography procedures: 1st : simulation. 2nd: 1st treatment and 2nd simulation. 3rd: 2nd treatment:

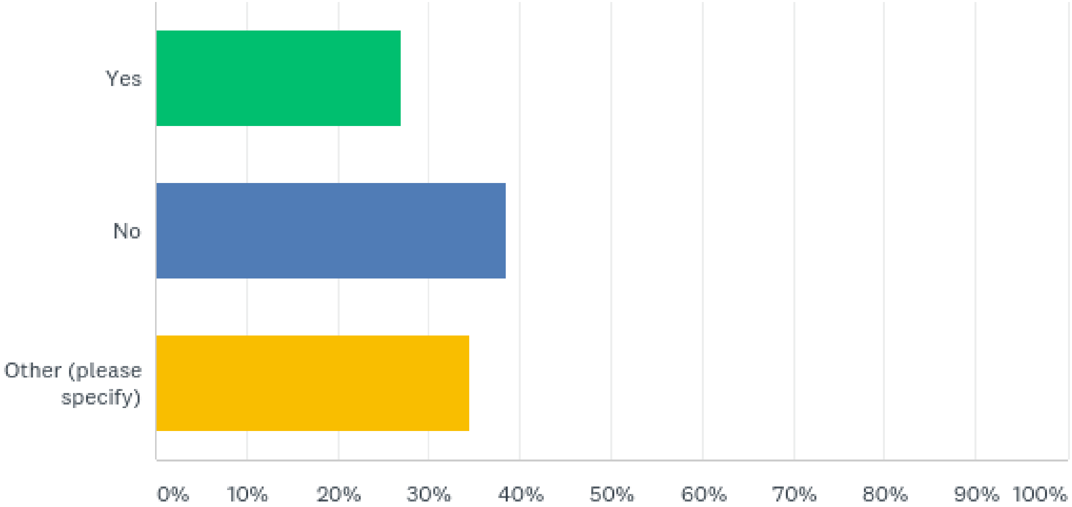

Q16: Do you recommend assessing the arterial liver anatomy before simulation?

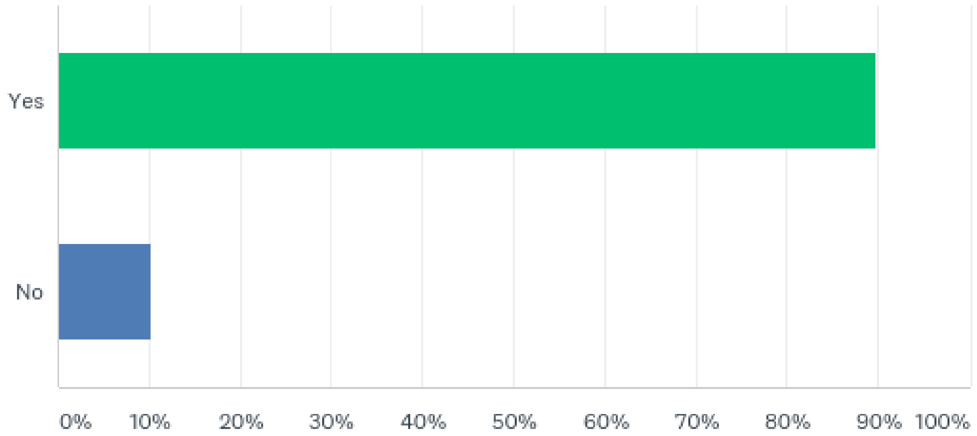

Q17: What is the rationale for the evaluation using 99mTc-MAA before SIRT?

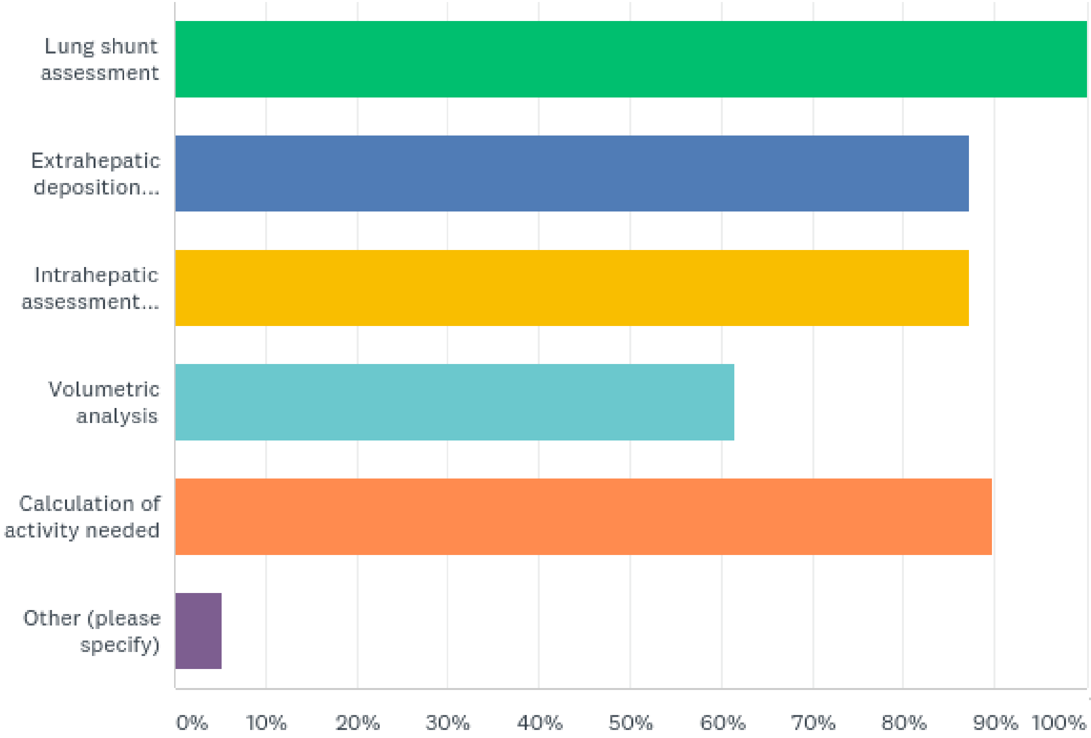

Q18: Which imaging method should be used to evaluate the lung-shunt with 99mTc-MAA?

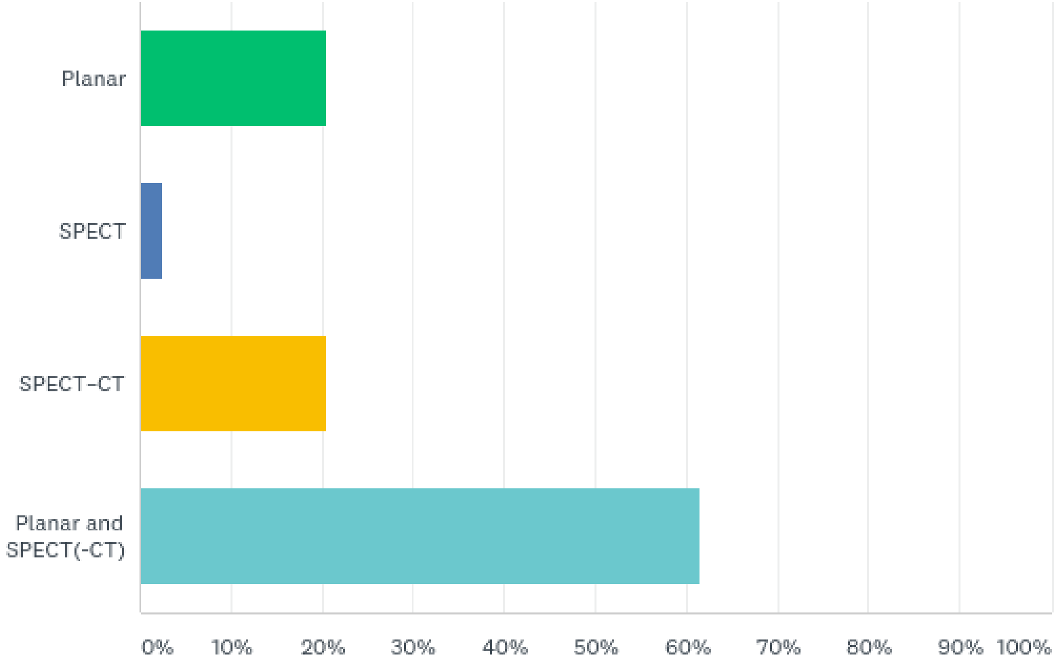

Q19: Which imaging method should be used to evaluate 99mTc-MAA distribution within the liver?

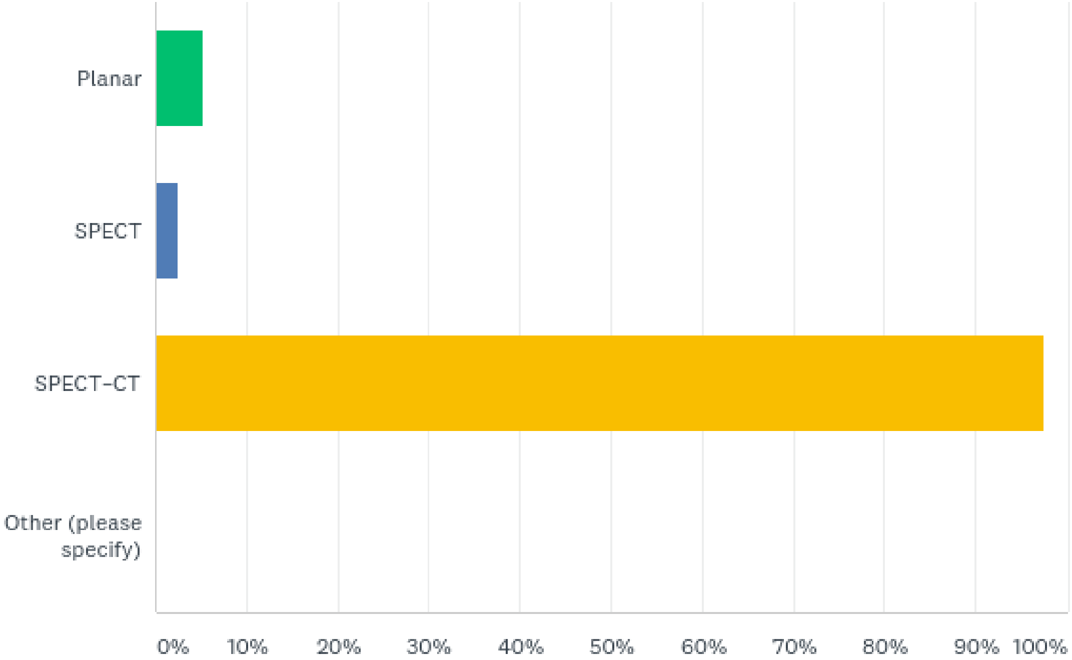

Q20: Should tumour(s) be directly delineated on 99mTc-MAA images?

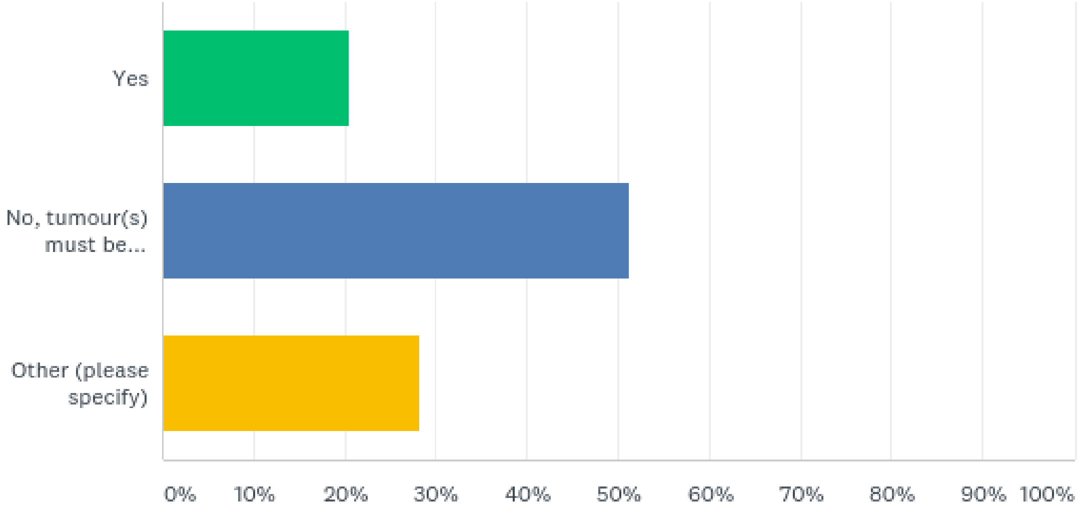

Q21: When there is less 99mTc-MAA uptake in the lesion than the healthy liver, should SIRT be withheld for that lesion?

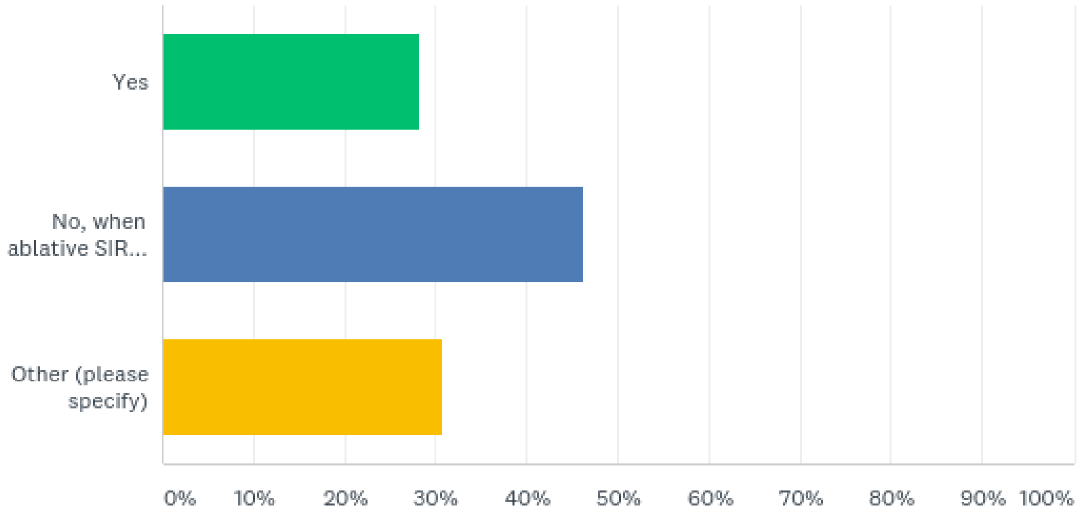

Q22: When should cone-beam CT be used for SIRT?

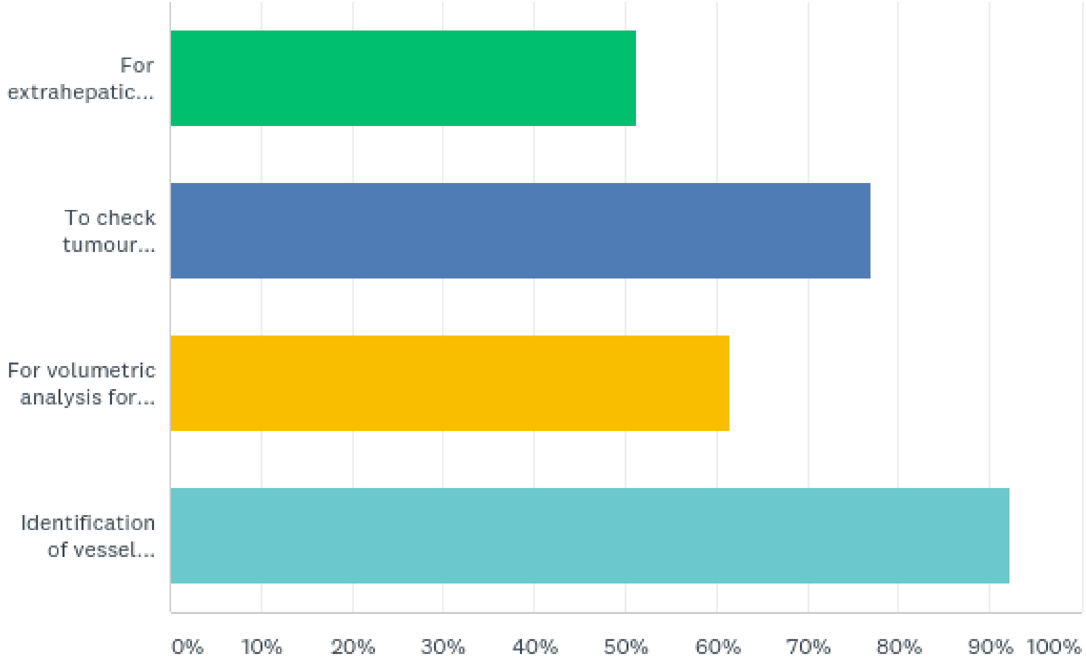

Q23: Which imaging method do you recommend for extra-hepatic deposition verification?

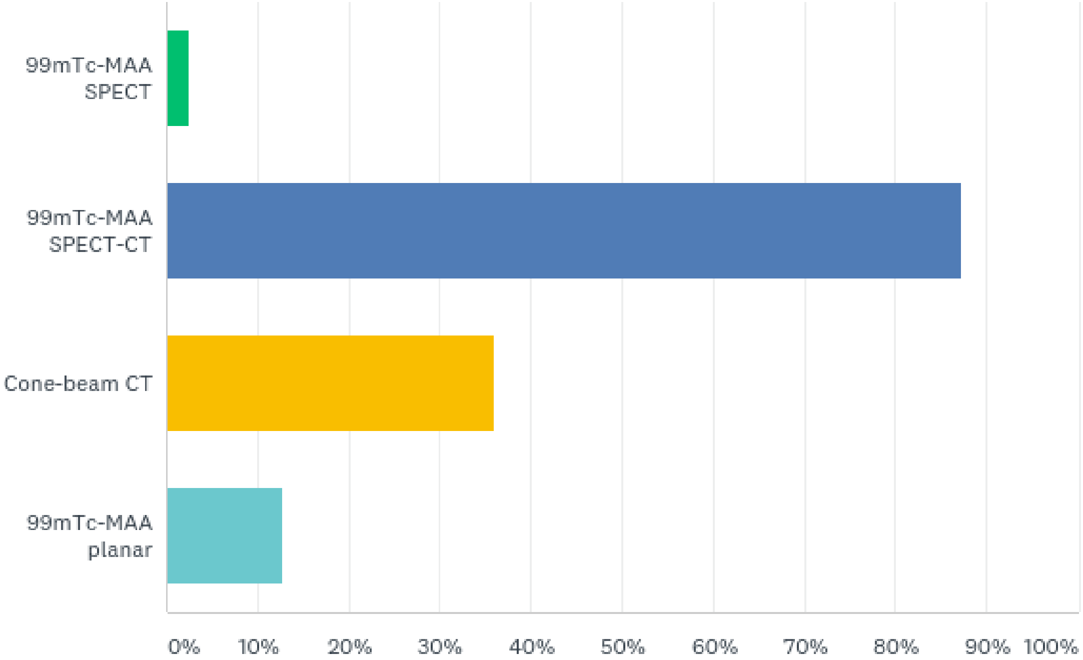

Q24: Which imaging method do you recommend for volumetric analysis?

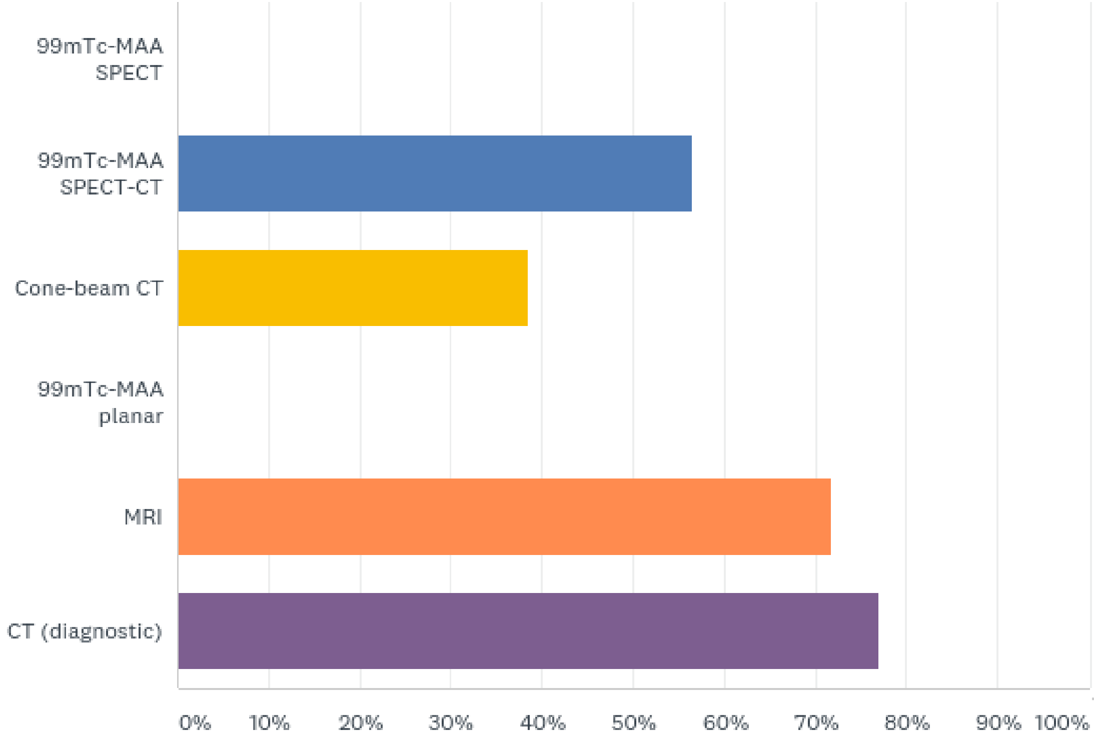

Q25: What is the maximum time one should allow between simulation and SIRT?

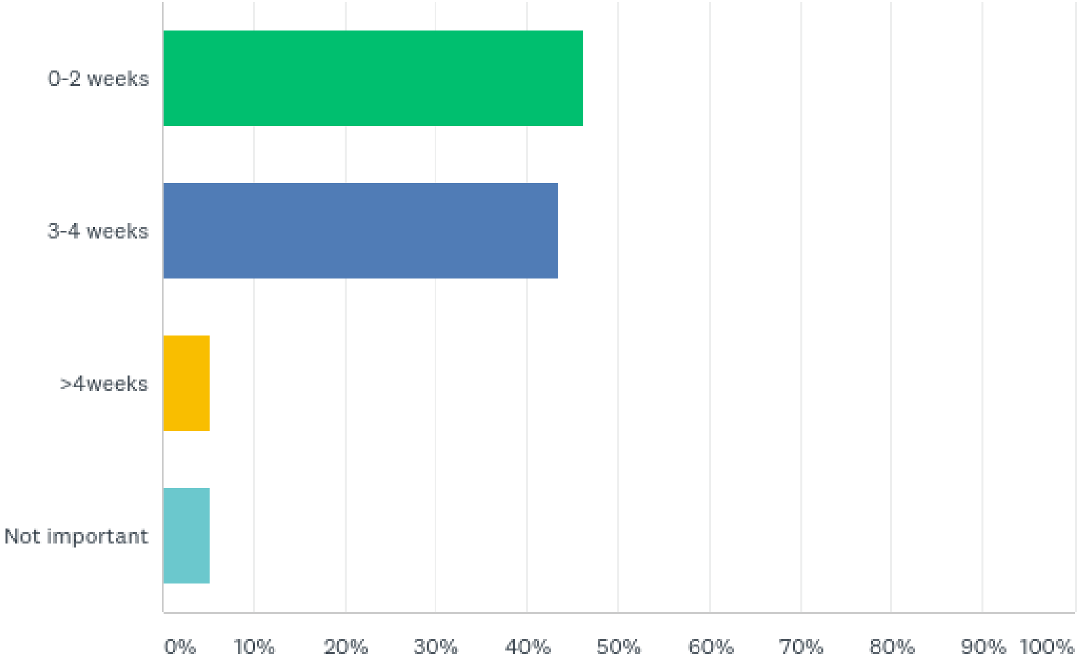

Q26: Should the necrotic portion of a tumour be included in the target volume?

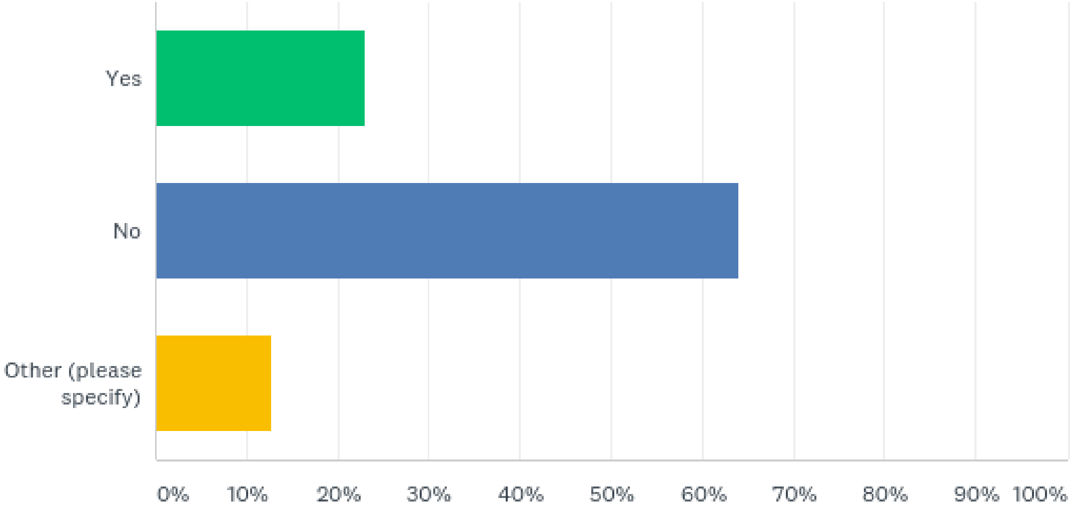

Q27: The simulation has been carried out, the activity to administer has been determined and the treatment is scheduled. For some reason the catheter position has to be changed. Would you recommend re-performing the simulation?

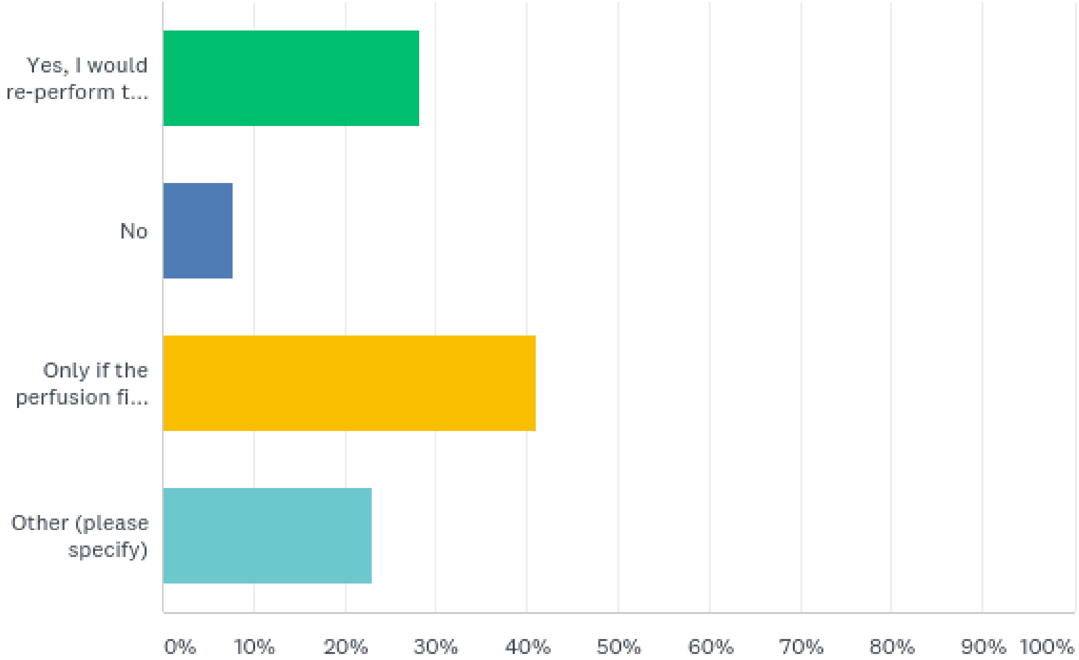

Q28: The multi-disciplinary tumour board decided to perform whole liver treatment: which activity prescription method do you recommend?

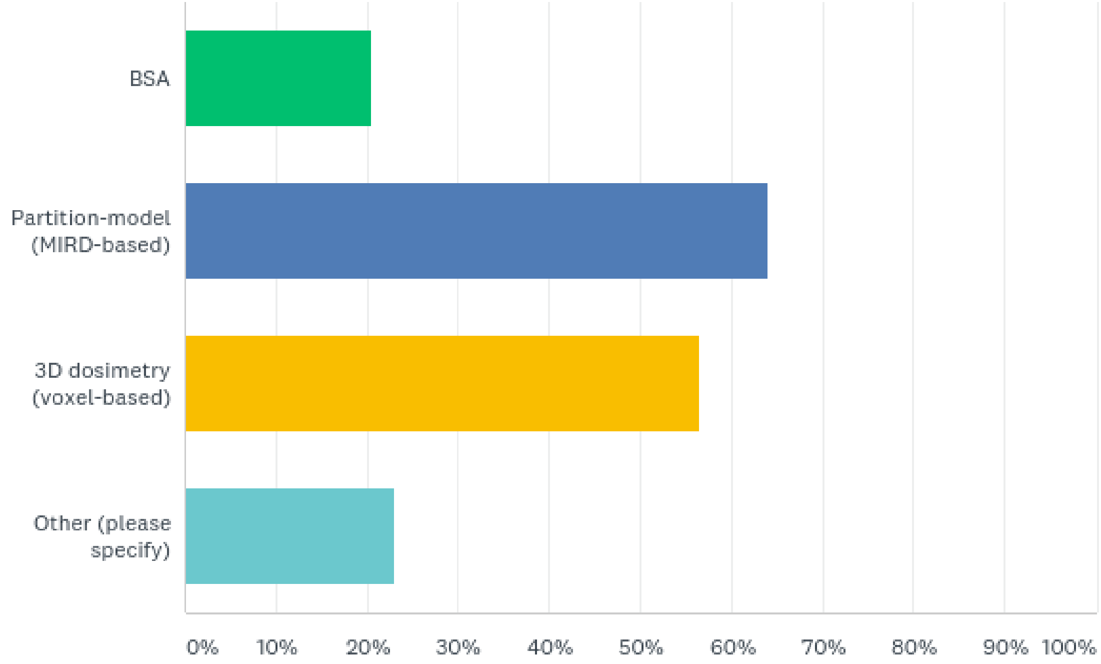

Q29: The multi-disciplinary tumour board decided to perform selective non-ablative treatment: which activity prescription method do you recommend?

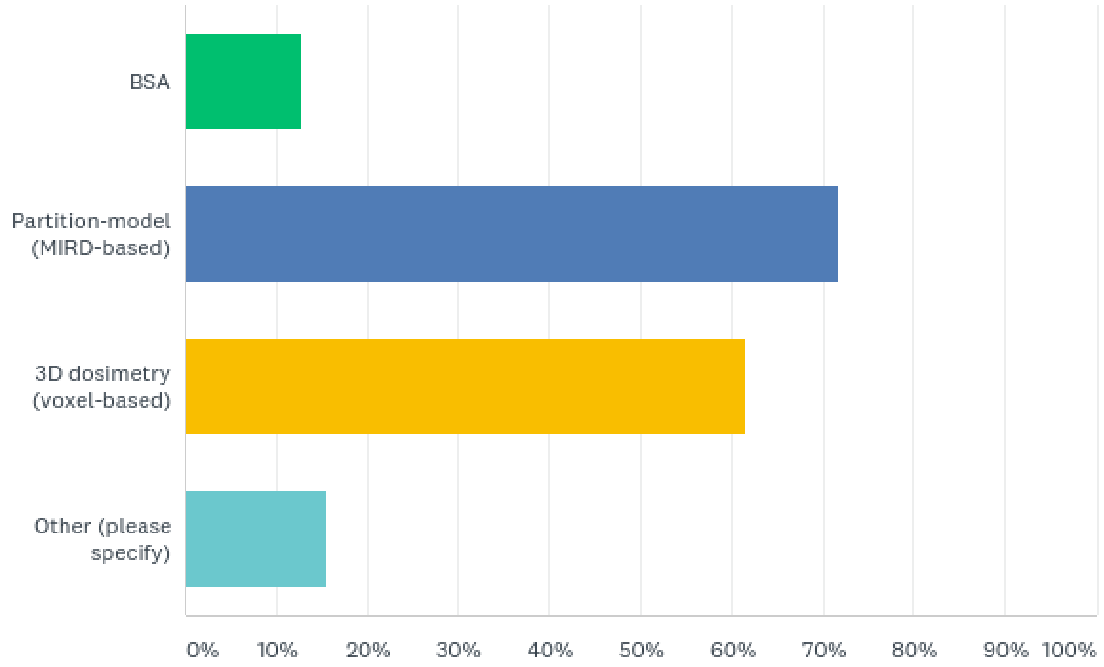

Q30: The multi-disciplinary tumour board decided to perform selective ablative treatment: is an activity prescription method recommended?

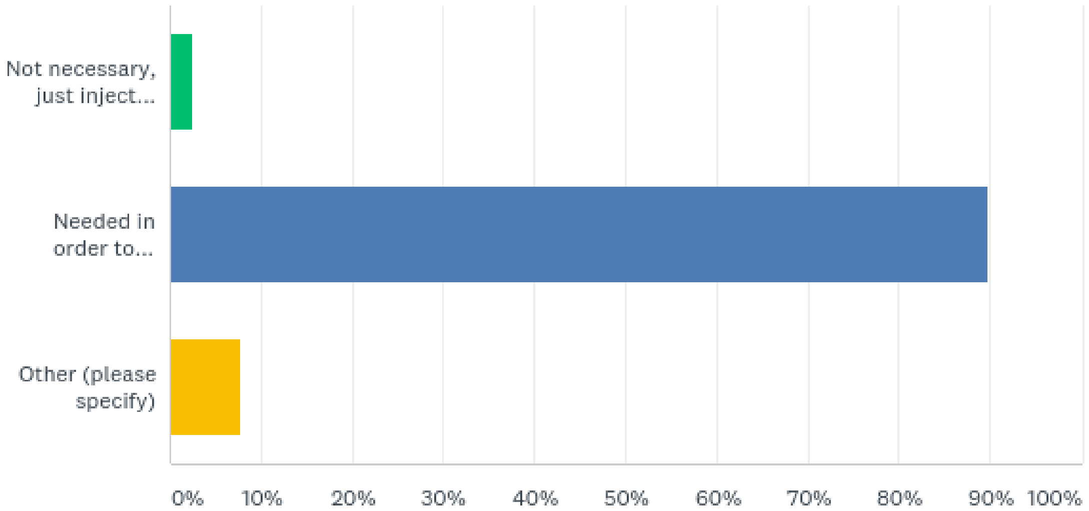

Q31: If needed: which activity prescription method do you recommend?

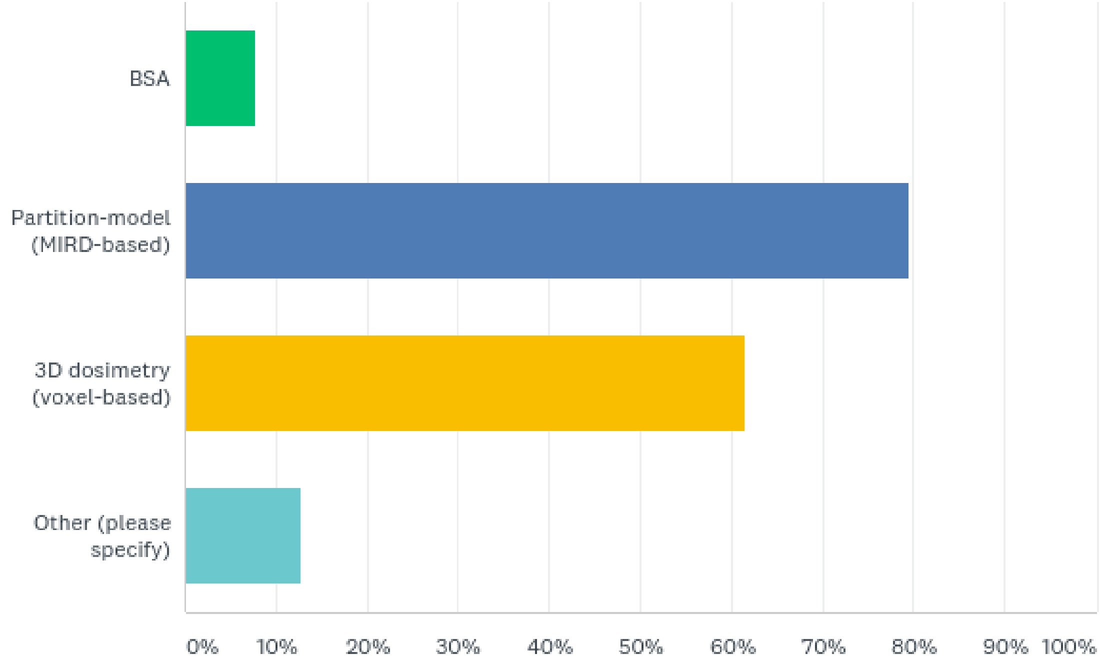

Q32: In which circumstances would you consider higher specific activity of resin microspheres (i.e. specific activity=activity per sphere; classical sphere specific activity is 50 Bq/sphere)?

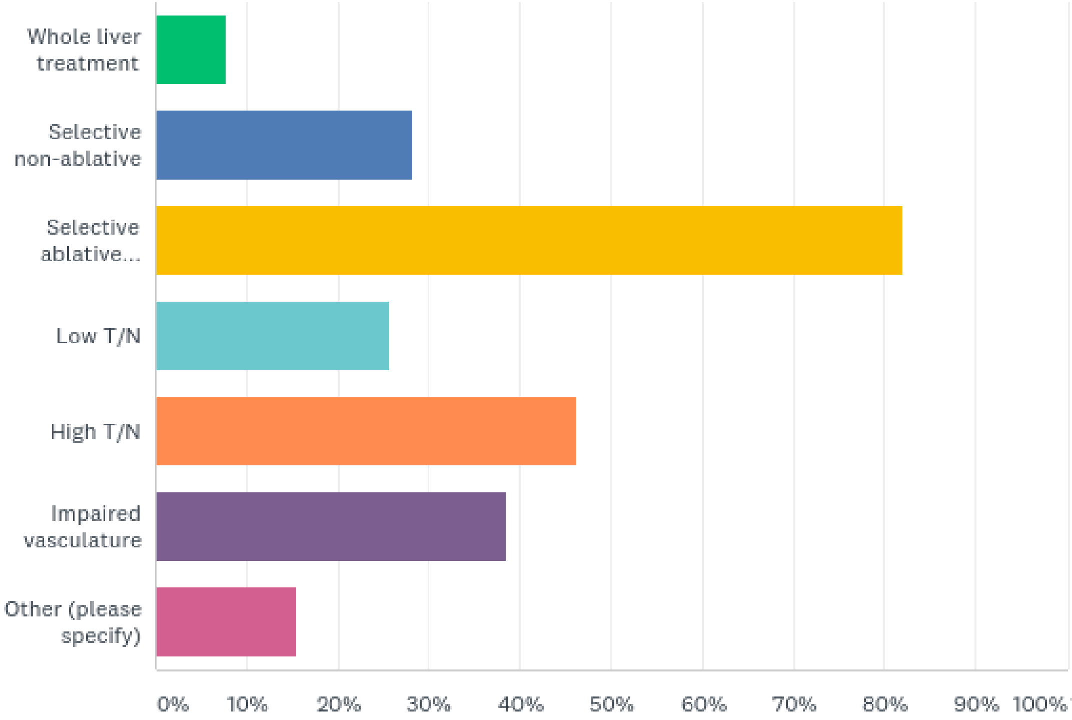

Q33: Should 99mTc-MAA images be used to determine the tumour to non-tumoural liver uptake ratio?

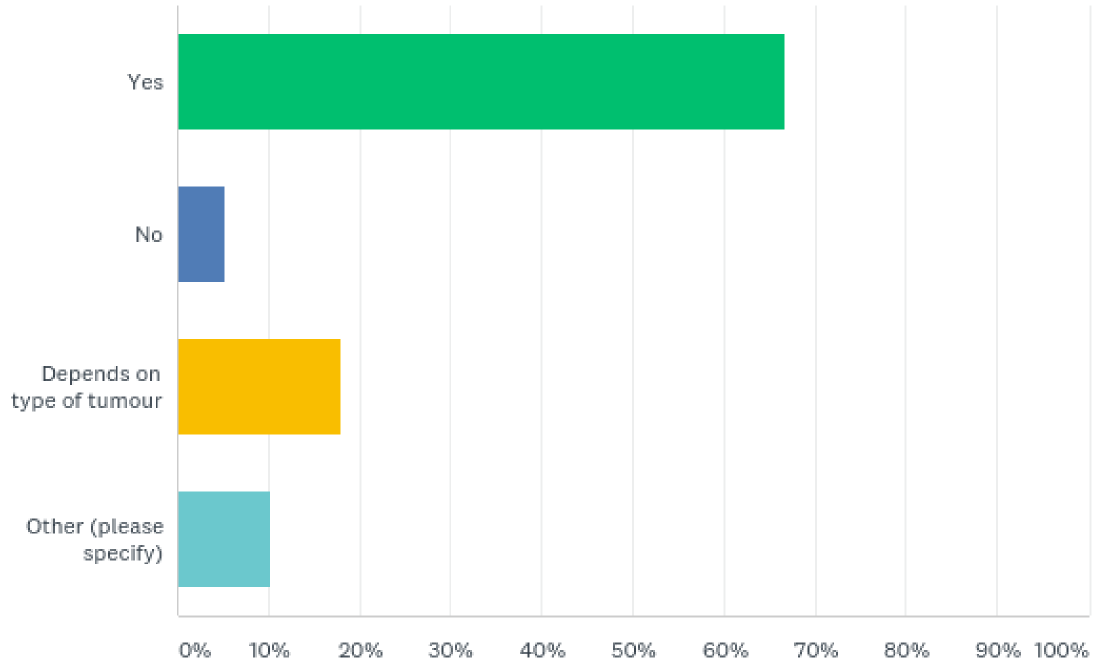

Q34: If you answered 'NO' to Q33: what imaging technique should be used to determine the tumour to non-tumoural liver uptake ratio?

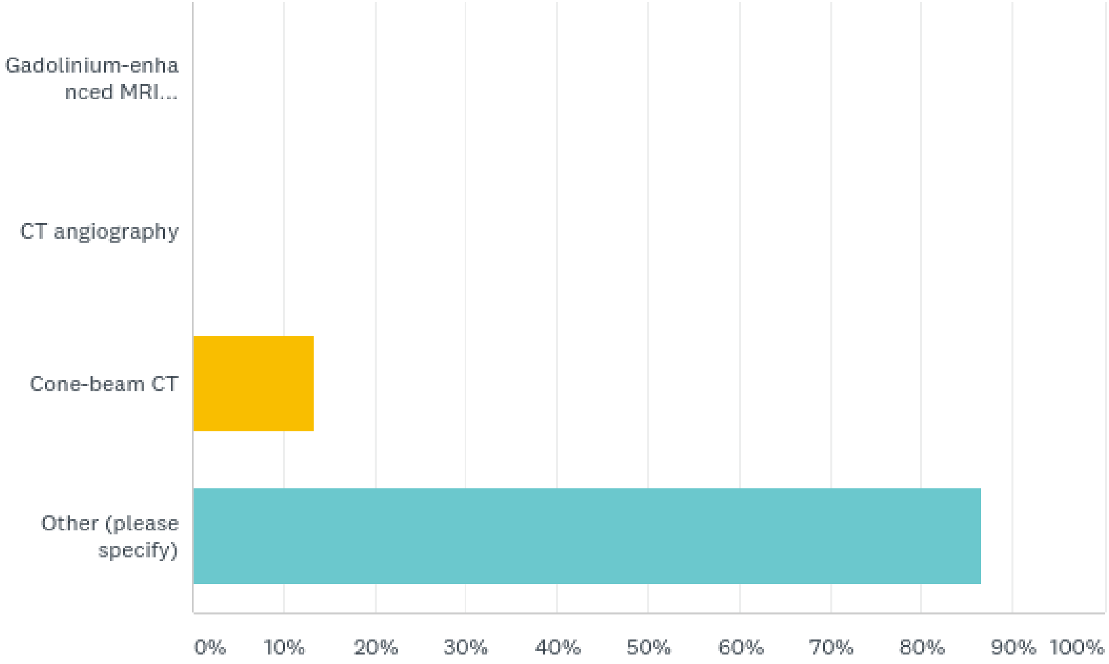

Q35: If you answered 'YES' to Q33: how do you recommend estimating the tumour to non-tumoural liver uptake ratio?

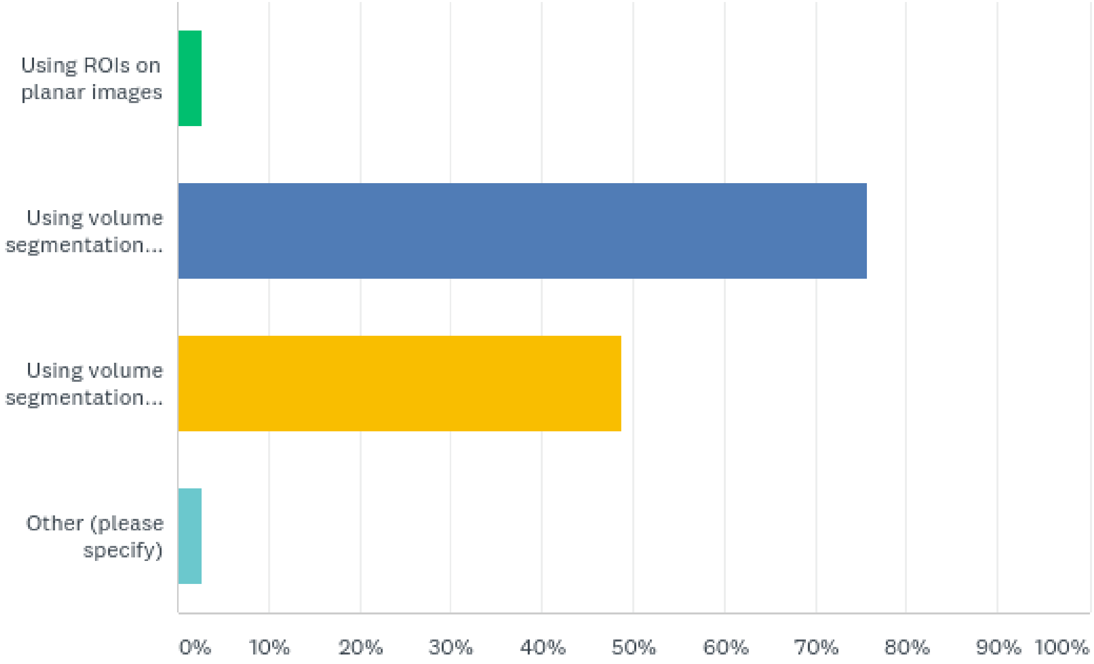

Q36: How should lung-shunt limits be expressed?

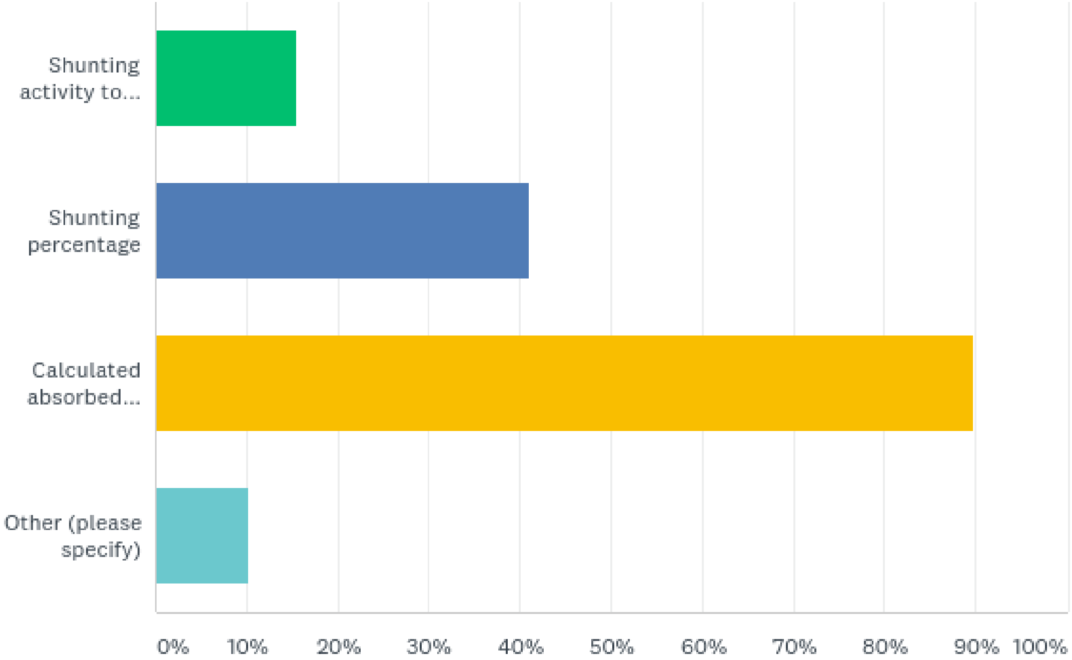

Q39: Would you recommend measuring the lung volume for assessing dose to lung tissue?

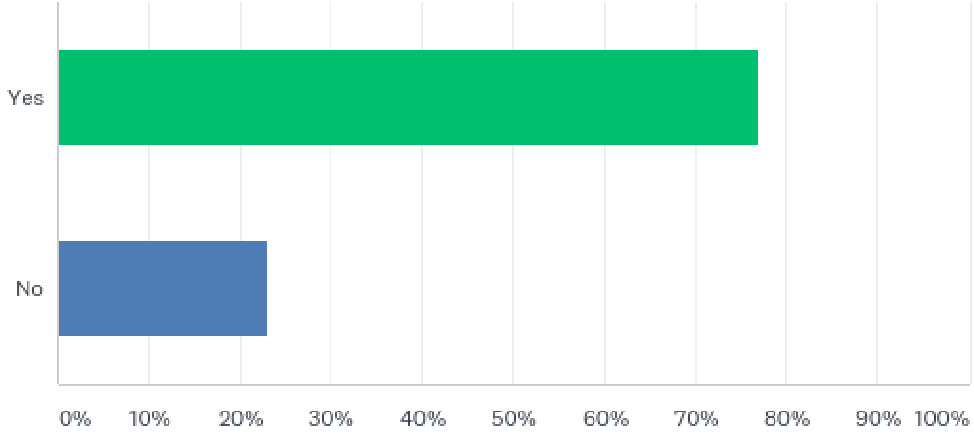

Q41: For uni-lobar treatment, would you use the same absorbed dose safety limits as for whole liver (bi-lobar) treatment?

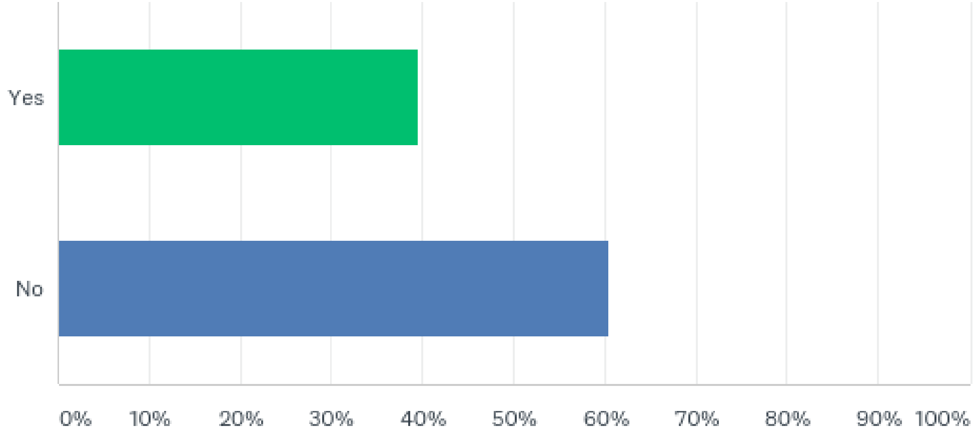

Q42: For uni-lobar treatment, would you perform a more aggressive treatment when the volume and function of the contralateral liver lobe is sufficient?

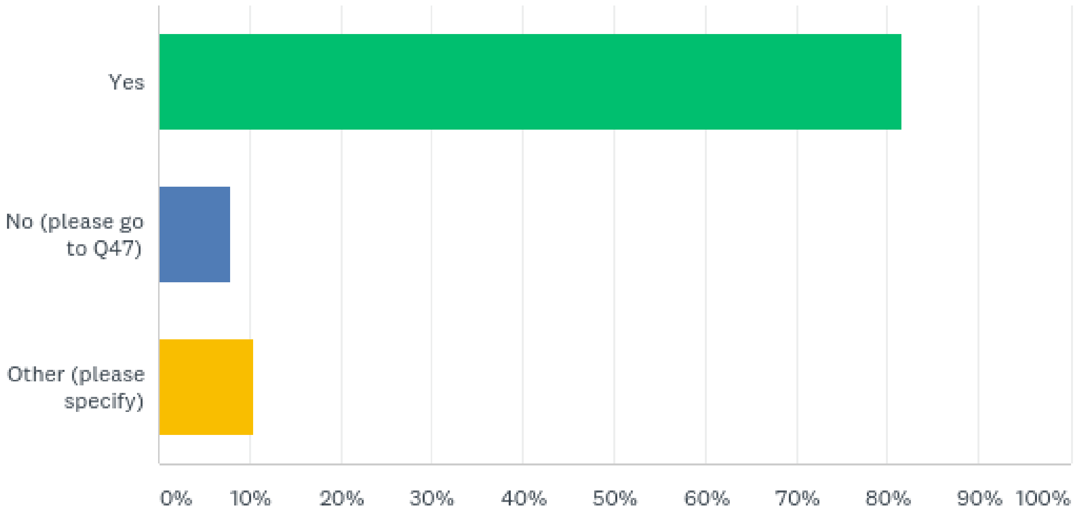

Q45: If you answered 'YES' to Q42: would you also perform a more aggressive treatment in cirrhotic patients?

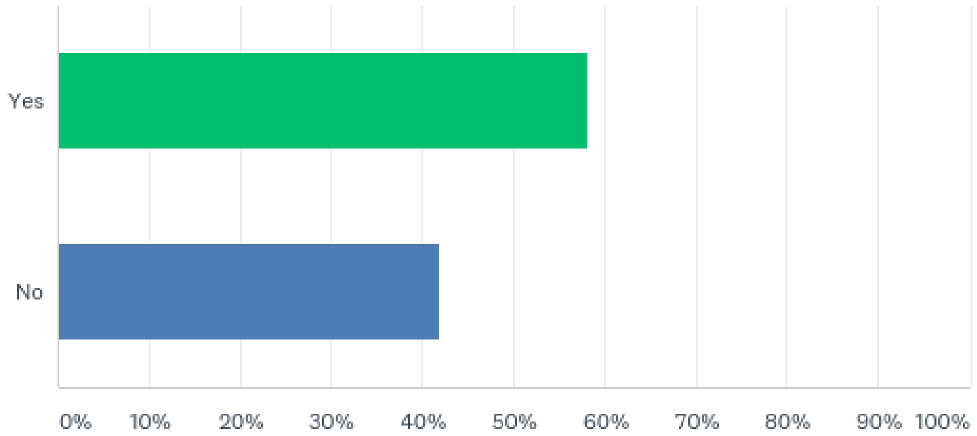

Q49: In such a pre-operative SIRT setting, what should be the minimal time window between SIRT and surgery?

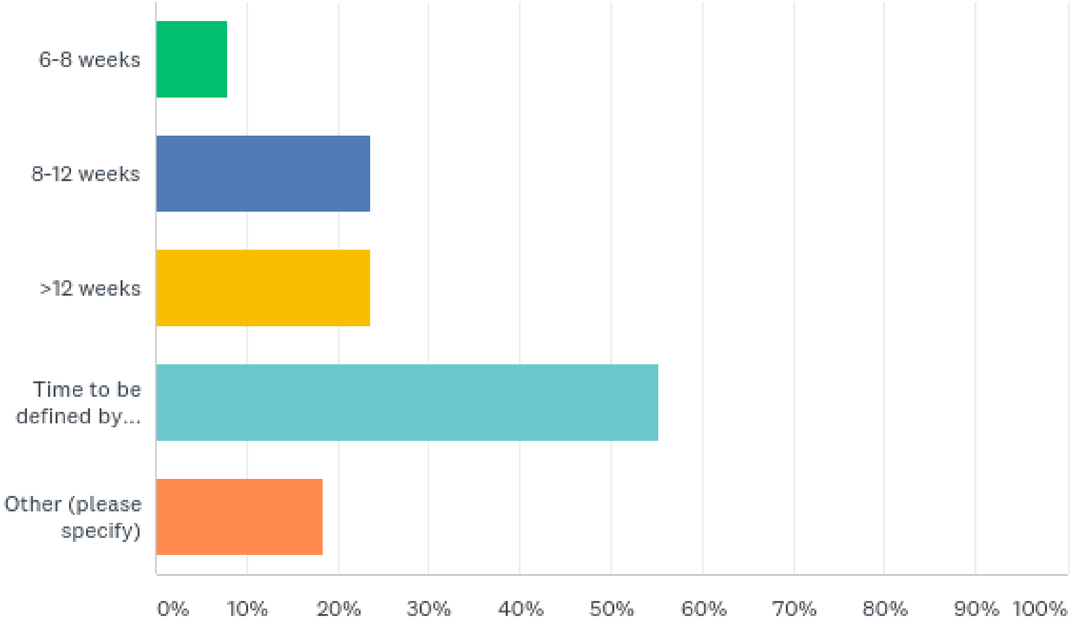

Q54: Do you think it is important to verify whether the position/location of the catheter is the same during SIRT as it was during the 99mTc-MAA simulation? If yes, how?

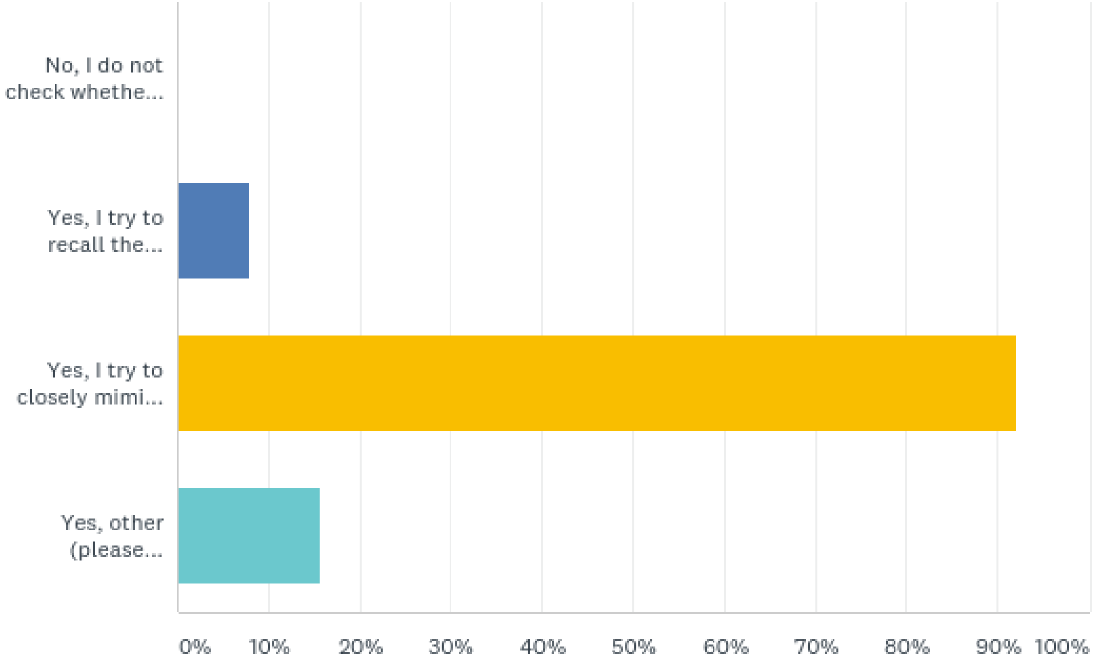

Q55: Would you determine the post-SIRT residual activity of microspheres in the vial, tubing system, syringe? If yes, how?

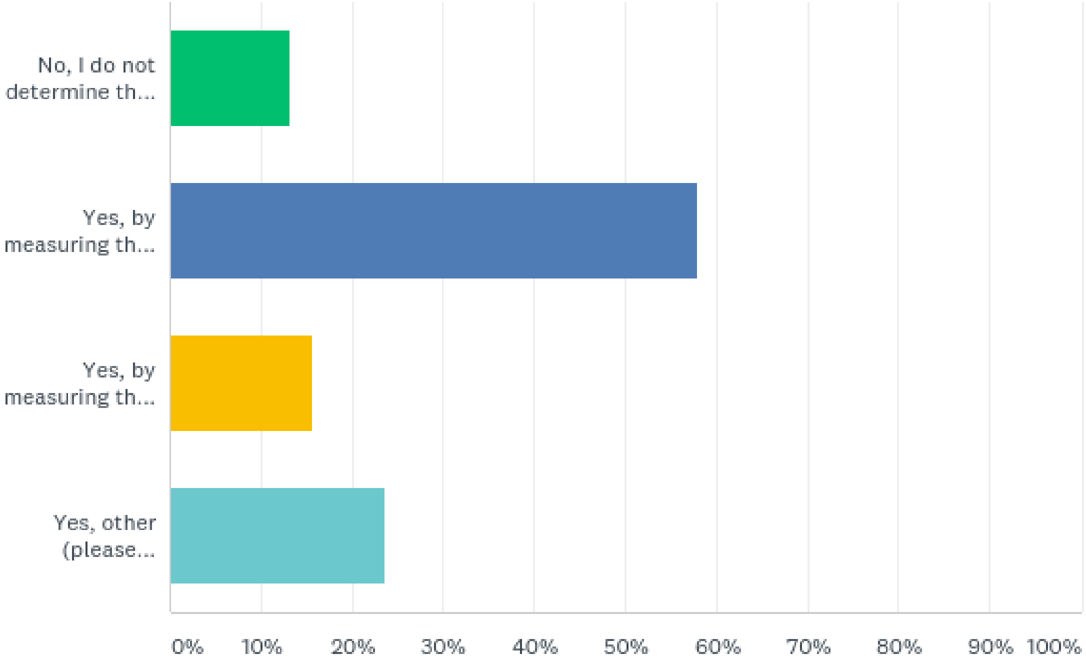

Q56: Post-SIRT imaging for treatment verification is used for:

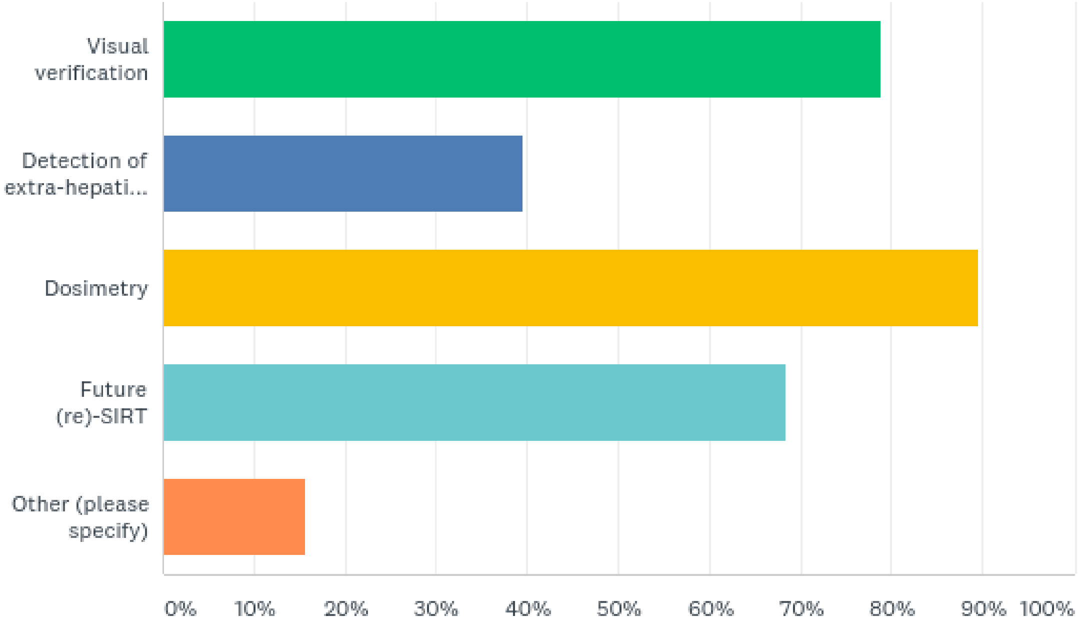

Q57: Post-SIRT imaging for treatment verification is:

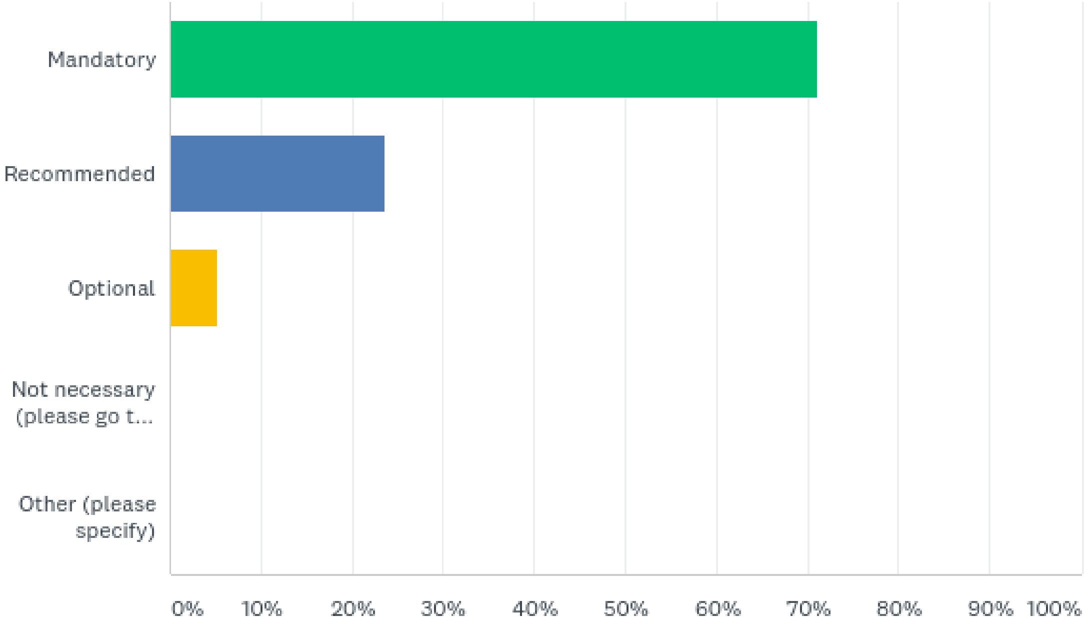

Q58: If recommended, treatment verification should be performed using:

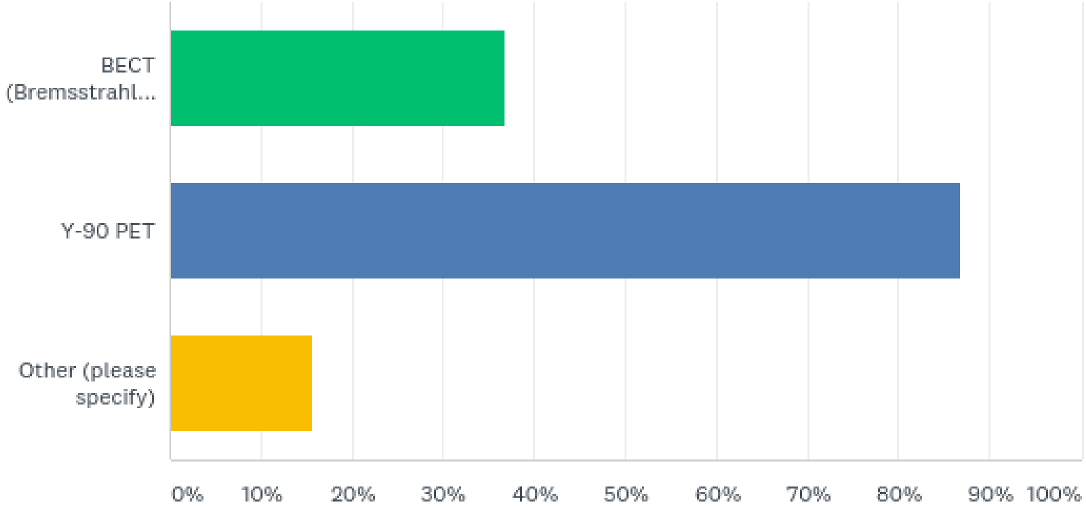

Q59: If recommended, treatment verification should be:

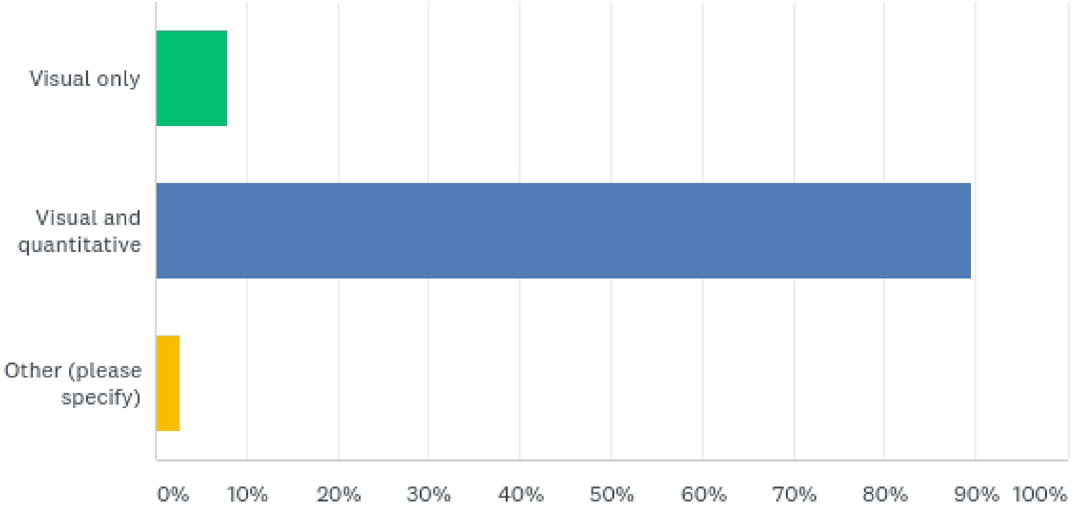

Q60: Post-SIRT dosimetry is:

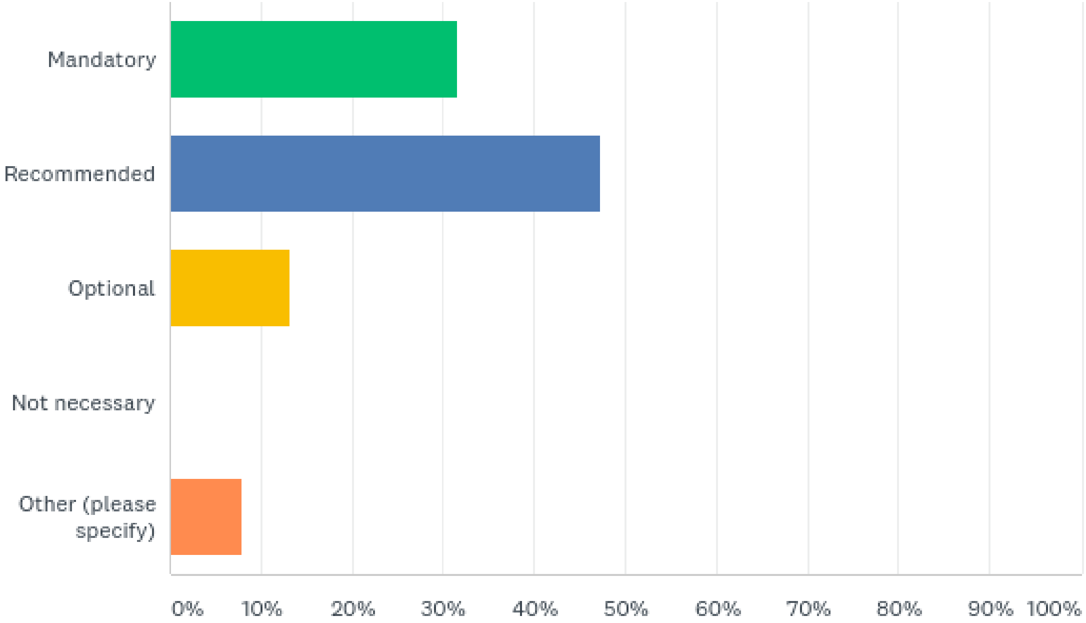

Q61: If post-SIRT dosimetry shows underdosing of the tumour, is retreatment recommended (to increase the cumulative dose to the tumour)?

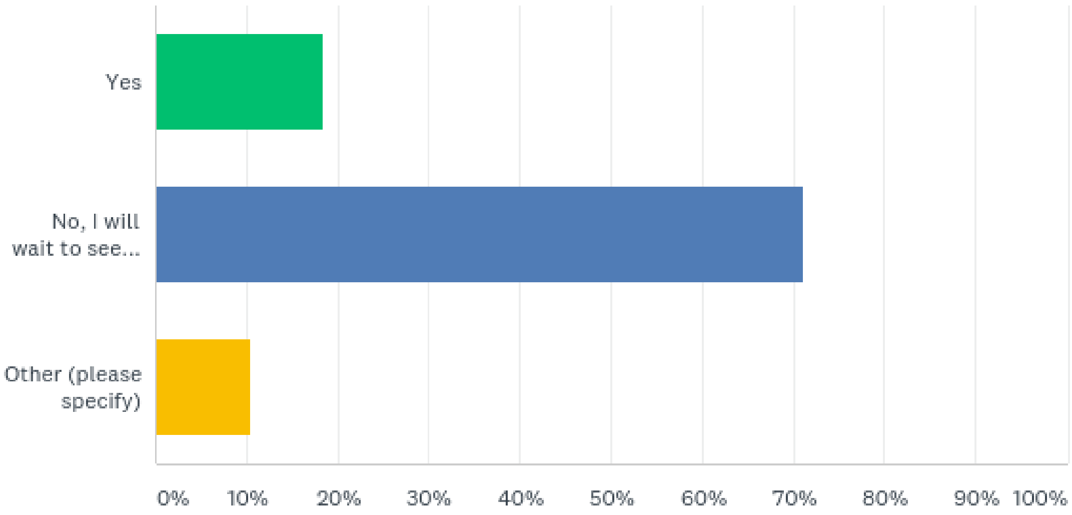

Supplement: Supplementary file 2 — SURVEY OF BEST PRACTICE -SIRT DOSIMETRY (PDF 449 kb) [file 259_2020_5163_MOESM2_ESM.pdf]
